# NanoDeNovo: De Novo Design of Anti-Poliovirus I Sabin Strain Nanobodies by Semi-Automated Computational Pipeline

**DOI:** 10.3390/ijms26199262

**Published:** 2025-09-23

**Authors:** Danil D. Kotelnikov, Katerina S. Tatarinova, Dmitry D. Zhdanov

**Affiliations:** 1 Institute of Biomedical Chemistry, 10/8 Pogodinskaya Str., 119121 Moscow, Russia; danil.kotelnikov.02@mail.ru; 2 Frumkin Institute of Physical Chemistry and Electrochemistry, Russian Academy of Sciences, 31-4, Leninsky Prospect, 119071 Moscow, Russia; tatakaterina@yandex.ru; 3 Higher Chemical College, Dmitry Mendeleev University of Chemical Technology of Russia, 9 Miusskaya Square, 125047 Moscow, Russia; 4 Department of Biochemistry, People’s Friendship University of Russia Named After Patrice Lumumba (RUDN University), 6 Miklukho-Maklaya Str., 117198 Moscow, Russia

**Keywords:** nanobody, Poliovirus I, Sabin, in silico, computational design, molecular docking, ab initio

## Abstract

Despite global vaccination efforts, poliomyelitis continues to cause paralytic cases, highlighting the need for alternative therapeutic approaches. Nanobodies offer significant advantages over conventional antibodies due to their small size, stability, and low immunogenicity, yet few have been developed specifically against poliovirus. This study presents a fully computational pipeline for de novo design of nanobodies targeting Virus Protein 3 (VP3) of the Poliovirus I Sabin strain. Our integrated approach employed Ig-VAE for scaffold generation, ProteinMPNN and RFantibody for sequence design, tFold-Ab/Ag for structure prediction, multi-platform molecular docking (Rfantibody, Rosetta3, ClusPro2, ReplicaDock 2.0), molecular dynamics simulations, and humanization tools. The pipeline identified three humanized nanobodies (scFv-0389-304-6H, scFv-0389-459-5H, and scFv-0743-166-7/H) that demonstrated strong binding to VP3 with binding free energies of −37.66 ± 10.35, −40.11 ± 20.01, and −48.62 ± 11.21 kcal/mol, respectively. All designs exhibited favorable physicochemical properties and high solubility. Notably, nanobodies humanized prior to CDR-loop design (scFv-0743-166-7/H) showed superior stability, binding affinity, and structural similarity to experimentally validated nanobodies. This work demonstrates the feasibility of a fully computational approach for designing promising nanobodies against viral targets, providing an alternative to traditional methods with potential applications in drug design.

## 1. Introduction

The global polio eradication program has resulted in a more than 99% reduction in cases caused by wild polioviruses [1]. However, vaccine-derived polioviruses still present paralytic outbreaks, leading to hundreds of cases in poorly immunized communities [2]. Despite the existence of numerous effective vaccines and global vaccination programs aimed at eradicating this disease, live-attenuated polioviruses can mutate and circulate in populations with inadequate immunization levels.

According to modern classification, the causative pathogen of this disease is poliovirus. It is an Enterovirus coxsackiepol and belongs to the Picornaviridae family, Ensavirinae subfamily, and Enterovirus genus. The development of antiviral agents for passive prophylaxis and diagnostics of poliomyelitis is still a relevant issue at this time.

Nanobodies are single-domain antibodies that consist of only the heavy antigen-binding region while missing their light-chain counterparts. They were first described in the serum of camels and sharks [3,4]. Due to the small size of HCAb (heavy-chain antibodies) up to 90 kDa in general and up to 15 kDa for nanobodies in particular [5,6], they are more suitable for production and use compared to the 150 kDa size of human immunoglobulin IgG. They are highly stable under certain denaturation conditions [7,8] and possess low immunogenicity [9,10].

The comparative advantages of nanobodies make them an attractive tool for fighting against infectious diseases [11]. They are not only useful for pathogen neutralization and vaccine design, but also for studying the structural biology of viruses, understanding the mechanism of virulence, protein engineering, and developing rapid diagnostics.

Nanobodies have been developed against a number of pathogenic viruses [12]; however, only a few of them have been specifically elaborated for poliovirus [13,14].

The traditional discovery and engineering of nanobodies often involve arduous and time-consuming experimental procedures, such as the immunization of camelids and subsequent selection from immune libraries [15]. In silico design strategies have emerged as a powerful alternative, enabling rapid pre-filtering of strong binders, broadening sequence diversity, and forecasting binding potency and selectivity [16,17]. This computational shift is part of a broader revolution in protein design, where predictive control over protein function is becoming increasingly feasible [18]. The integration of machine learning and molecular modeling techniques has been pivotal in refining the efficacy of nanobodies by expanding the sequence and chemical space beyond that typically explored by traditional methods. Such approaches leverage sophisticated algorithms and computational power to model complex biological interactions, predict structural characteristics, and optimize functional properties, thereby significantly reducing reliance on extensive in vitro screening and accelerating the development pipeline for these promising biologics. This includes advancements in areas like affinity maturation and the identification of unique binding mechanisms [19,20], but lack an all-in-one approach capable of unifying a structure–sequence de novo design for target-specific nanobodies.

The aim of this paper was the de novo design of nanobody structures specific to the Poliovirus I Sabin strain by an integrated semi-automated reproducible computational approach utilizing all recent advances in nanobody in silico design.

## 2. Results

### 2.1. Sequence Sampling and Structural Quality Control

The overall pipeline workflow started with the generation of 1000 side chain-free nanobody scaffolds by Ig-VAE followed by ProteinMPNN sequence sampling. All of the sequences were refolded by tFold-Ab and aligned against scaffolds to ensure the structural integrity of new designs.

**Figure 1:**
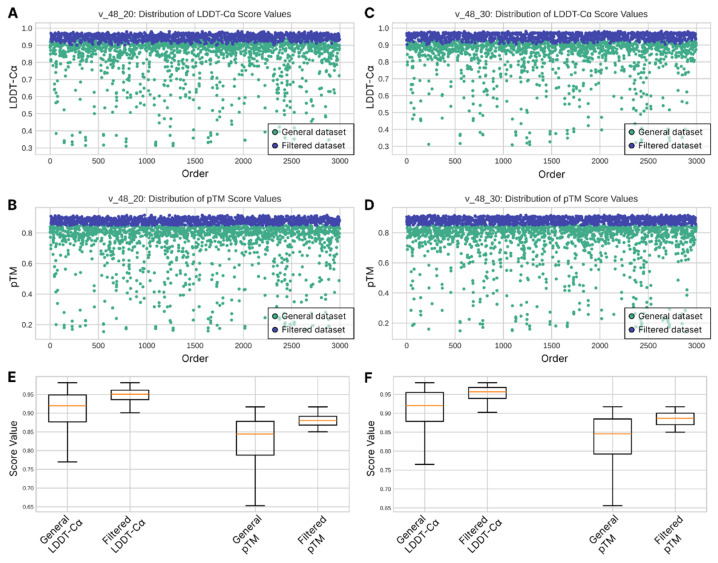
(**A**–**D**) Distribution of LDDT-Cα and pTM scores values of tFold-Ab-folded models of v_48_020 and v_48_030 datasets (green dots represent general dataset, blue dots ― filtered dataset, based on thresholds, described in Section 4). Scatter plots depict that the filtration stage has a significant impact on the quality of all of the nanobody models, taken as further inputs either for the alignment stage or molecular docking. (**E**,**F**) Descriptive statistics of structural quality of designed nanobodies for v_48_020 (**E**) weight model dataset group and v_48_030 (**F**) weight model dataset group (calculated only for LDDT-Cα score). The data show that the v_48_030 weight model group has a slightly better overall quality of structures, given the same initial scaffold.

The general dataset of all folded nanobody structures resulted in the following stats: (a) LDDT-Cα min 0.3103, max 0.9813; (b) pTM min 0.1537, max 0.9168. After filtration, 1392 and 1417 structures out of 3000 for the v_48_020 and v_48_030 weight model dataset groups correspondingly resulted in satisfactory LDDT-Cα and pTM scores (Figure 1A–D). Overall structure quality assessment based on LDDT-Cα score both before and after filtration demonstrates slightly better values for the v_48_030 model produced sequences. The basic descriptive statistics for the v_48_020 vs. v_48_030 datasets before filtration are (a) a mean of 0.887 < 0.893 (the higher the better), (b) a median equal to 0.920 for both (the higher the better), and (c) a standard deviation of 0.108 > 0.104 (the lower the better); after the filtration process, the values are (a) a mean of 0.948 < 0.953, (b) a median of 0.95 < 0.957, and (c) a standard deviation equal to 0.017 for both (Figure 1E,F).

**Figure 2:**
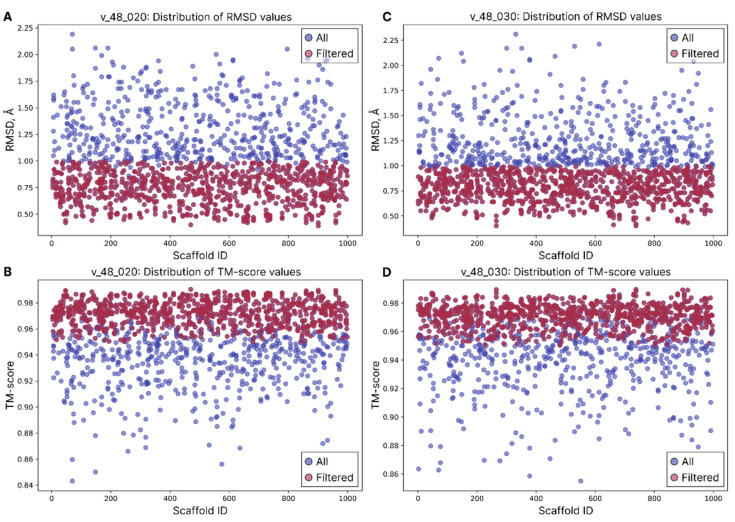
Distribution of RMSD and TM-score values among v_48_020 (**A**,**B**) and v_48_030 (**C**,**D**) weight model group structures of quality filtered nanobodies. Blue dots represent the overall filtered sample (same as on Figure 2), while red dots represent successfully aligned folded nanobodies with their corresponding Ig-VAE scaffolds (thresholds: RMSD < 1.0 Å and TM-score > 0.95).

The alignment-based filtering stage resulted in the following outcomes (Figure 2): for the v_48_020 model weight group, 1340 out of 1382 structures were passed; the average RMSD and average TM-score values are 0.9686 Å (Figure 2A) and 0.9574 (Figure 2B) correspondingly. For the v_48_030 model weight group, 1378 out of 1417 structures were passed; the average RMSD and average TM-score values are 0.9558 Å (Figure 2C) and 0.9591 (Figure 2D) correspondingly. Because the majority of structures passed the basic structure alignment filtering criteria, we attempted to perform two additional rounds of quality filtration by tweaking RMSD to <1.5 Å in the second round, and <1.0 Å in the third one; the TM-score threshold was increased to 0.95 in both extra rounds.

After the second and the third rounds of the filtration process, the number of passed structures decreased significantly: 838 out of 1382 nanobodies for the v_48_020 model weight group and 905 out of 1417 for the v_48_030 model weight group. The whole filtration process ended up with 1743 structures in total, which were taken as the most successful structures for the further virtual screening experiment.

### 2.2. Nanobody–VP3 Complex Virtual Screening

**Figure 3:**
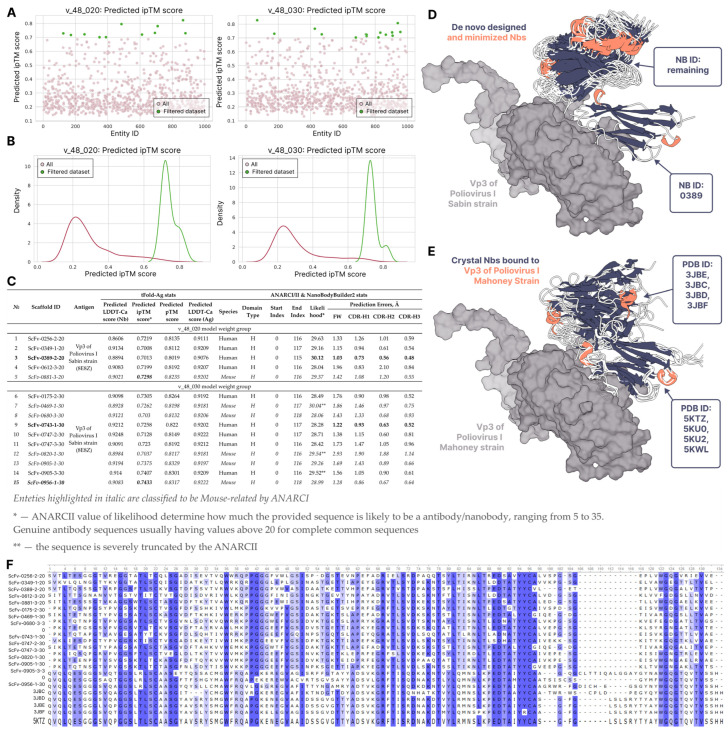
(**A**) Scatter plot of nanobody entities passing (green) the filtration process against those that are non-passing (pink) based on the ipTM score value (0.7), chosen as a key limiting factor in Nb–VP3 complex structure prediction, depending on the ProteinMPNN model used in the sequence design. (**B**) KDE graphs built on the same data represent that the v_48_030 model-derived sequences have higher densities, resulting in a higher number of successful structures of Nb–VP3 complexes compared to v_48_020-derived ones. (**C**) Table of all designed nanobodies identified as H-chains by ANARCI and whose structures were successfully predicted by NanoBodyBuilder2. Nanobody scaffolds scFv-0389-2-20 and scFv-0743-1-30 are highlighted in bold as the most accurate from each of the datasets based on the NB2 predicted errors in the framework (FW) and the CDR-H1, H2 and H3 regions. (**D**,**E**) Structural evaluation of predicted complexes of VP3 (Poliovirus I Sabin strain) (shown in surface) with de novo-designed nanobodies (shown in cartoon) compared to the crystal structures of experimentally validated nanobodies bound to VP3 (Poliovirus I Mahoney strain) revealed near identical patterns of their rigid body placement by deep learning approach (nanobodies are colored based on secondary structure, white – loops, blue – sheets, orange – helices). (**F**) Multiple sequence alignment of de novo-designed nanobody sequences with sequences of crystal nanobody structures available in RCSB-PDB. The highlighting is based on the conservation levels of sequences with the threshold of 50%. MSA reveals a high consistency level in the FW regions of the de novo-designed and folded scaffolds, only lacking antigen-specific CDR information.

After selection of the most successful nanobody structures with newly designed sequences, we conducted a virtual screening experiment of the best candidates capable of binding to VP3 via the tFold-Ag package. The obtained results included 104 mid-confident complex structures with ipTM > 0.6 39 from the v_48_020 dataset and 65 from the v_48_030 dataset. Further, according to the Section 4, we increased the bottom threshold of ipTM up to 0.7, resulting in 27 more mid-confidence complexes (Figure 3A,B).

The ANARCI renumbering and domain determination process identified nine nanobody structures to be light-chain-like (five from the v_48_020 dataset and four from v_48_030), one nanobody structure was not recognized by ANARCI at all, and two failed to be predicted by NanoBodyBuilder2 for an unknown reason. The overall validated pool of nanobodies consisted of 15 structures (Figure 3C).

Because the tFold-Ab/Ag model outputs the structures of complexes with clashes and unrealistic C-Cα, C-O, and C-N interatomic bond lengths, thus resulting in different structural artifacts and possibly unfavorable side-chain conformations, we performed an energy minimization step via OpenMM (as implemented in NanoBodyBuilder2, as well). All of the further steps of cross-validation and different evaluation approaches, encompassing tFold-Ag complexes, make considerations using these minimized structures.

The visual analysis of energy-refined complexes with available crystal VHH-domain antibodies (Figure 3D,E) showed that the designed structures target the VP3 protein in the same pattern. This assessment can serve as an instance of a successful nanobody design process because we did not attempt to validate our results by in vitro experiments in the present study or use a computational CAPRI-Q assessment tool, due to large differences in complex structures and in sequences overall. The sequence completeness of the designed nanobodies was inspected in the Unipro UGENE (ver. 52.1) software [21] by MUSCLE multiple sequence alignment [22] with the same crystal nanobodies (Figure 3F). It is shown that although the nanobody binder produced is the combination of Ig-VAE and the ProteinMPNN design, possessing a complete structure and sequences with successfully identified CDR-H loops and consistent framework regions by NB2 and low predicted errors, it is not as long as classically produced nanobodies during in vitro experiments, as depicted by some missing residues at the beginning of the sequence and non-antigen-specific CDR-H loop regions (Figure 3F).

### 2.3. Antigen-Specific CDR-Loop Design

To address the issue of the non-antigen-specific CDR-loops of the best scaffolds, shown in Figure 3C, we extended the design process with the new RFantibody pipeline, which we included in our main design process with the purpose of redesigning all of the CDR regions and solving two problems: insufficient length of the design structures and non-antigen-specific design of CDR-loops in previous stages. The RFantibody sampling strategy consists of three consecutive steps: (1) “blind” docking with dummy CDR-loops (using the fine-tuned RFdiffusion model), (2) CDR-loop sequence sampling (using ProteinMPNN), and (3) re-docking of complete nanobody structures with the antigen (using the fine-tuned RoseTTAFold2 model).

The antigen-specific CDR design yielded an overall pAE and ipAE value range of ~2 to ~10. It was originally recommended to filter out successful designs with pAE < 10, which includes an unrealistically wide range of possible nanobody candidates (5000 designs for each scaffold in our case). PAE/ipAE scores were introduced relatively recently within AlphaFold-Multimer release [23] and lack versatile open-source benchmarks that establish baseline values, which can be considered as a reliable indicator of successful design. However, several other studies [24,25], including Evans et al. [23], state that lower values are preferable, particularly when paired with other validating metrics such as pLDDT, pTM/ipTM, and different energy functions (such as Rosetta ddG, etc.) or calculated metrics (SAP score, number of H-bonds, etc.) obtained from docking experiments. In tandem with the previously mentioned studies, the RFantibody approach, according to its Section 2, and Zhang et al.’s recent Ras-binder design approach [26], set the pAE baseline cutoff at 5.0 Å paired with pLDDT > 0.9, considering the rest of the designs unsuccessful. From our perspective, we modified the design filtering criteria slightly and distributed all of the possible designs into three main clusters. High-quality predictions were considered to have pAE < 4, medium-quality models ranged from 4 ≤ pAE < 7, and acceptable-quality models had pAE ≥ 7. From a rational standpoint, successful designs have narrower thresholds in our approach. These thresholds are intended to minimize the potential off-target effects of nanobodies and are described with both pAE and ipAE. This eliminates the need to attribute individual cutoffs as these values are length-dependent: ipAE is always less than pAE. At the same time, we provide mid- and acceptable-quality groups, as was introduced in the ipTM case during virtual screening, that serve as a “grey zone” that can be utilized in several ways: (1) as a reserve pool of designs in cases where overall design results in zero high-quality structures that must undergo obligatory cross-validation (docking, alignment, and molecular dynamics calculations); (2) to indicate a need to change the input scaffold; (3) as a hallmark to reset design and sampling parameters, such as the number of docking poses, number of sampled sequences (overall sampling size), number of connections, and CDR lengths.

**Figure 4:**
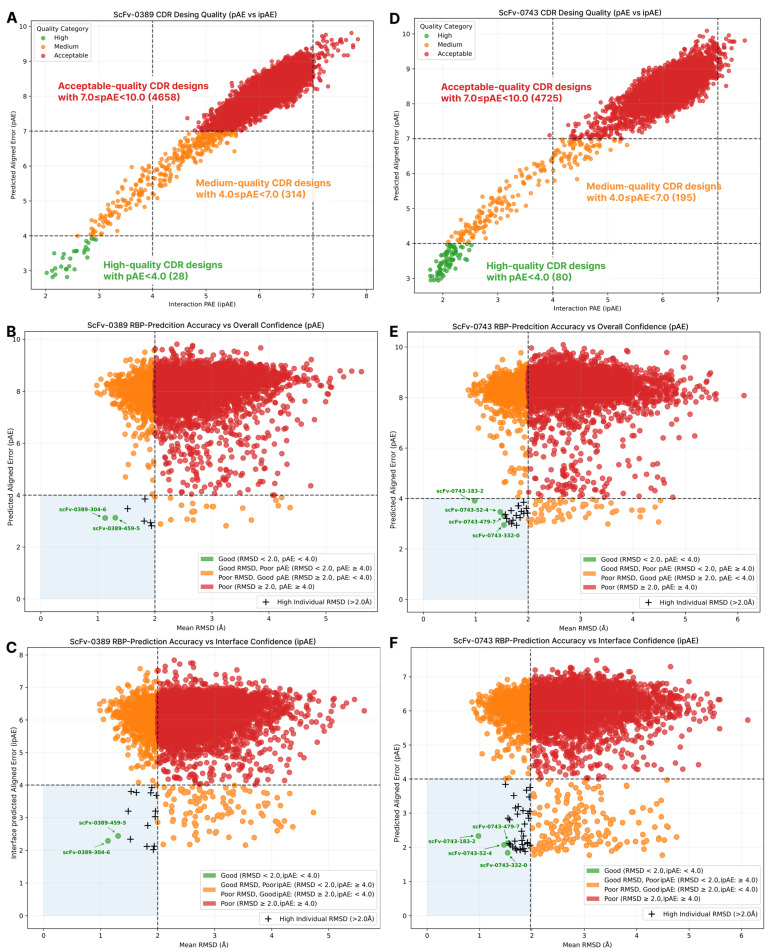
Scatter plots illustrating the design results of VP3-specific CDR H1-H3 loops for scaffolds scFv-0389 (**A**–**C**) and scFv-0743 (**D**–**F**). Scatter plots (**A**,**D**) depict the quantitative relationships between clusters differentiated by pAE scores, where high-quality models have pAE < 4, mid-quality models range from 4 ≤ pAE < 7, and acceptable-quality models have pAE ≥ 7. Scatter plots (**B**,**C**) and (**E**,**F**) clarify the relationships between pAE and ipAE scores and the RMSD_mean_ value (calculated as the RMSD of the framework and H1-H3 regions aligned with the target). Models with RMSD_mean_ < 2.0 Å, but with an RMSD > 2.0 Å for any individual region, spotted with “+” symbols, were not considered successful in the present study.

The quantitative composition of these clusters resulted in 28/314/4658 designs for scFv-0389, and 80/195/4725 designs for scFv-0743 of different qualities, where the relations of the high-quality designs to the total amount were 0.56% and 1.6%, correspondingly (Figure 4A,D).

As was mentioned earlier, a set of RMSD values is the second relevant criterion for structural integrity assessment. We filtered all of the pAE/ipAE-validated designs in two steps. First, we calculated the RMSD_mean_ of the framework-aligned RMSD_antibody_, RMSD_CDR_, and RMSD_H1/H2/H3_, setting the filter at <2.0 Å (green blue quadrant on scatter plots, Figure 4B,C,E,F). Second, we checked if any of the values used in RMSD_mean_ calculation could possibly exceed 2.0 Å inclusively (“+” spots on scatter plots, Figure 4B,C,E,F) and excluded those designs from the successful pool. As was intended by the developers of RFantibody software, the main idea behind this particular filtration is that if the binding mode and the quality of the predicted structures are reproduced coherently between steps one and three of the CDR design process, as established with the pair of both pAE/ipAE and RMSD values, and cross-validated via external computations, such as docking or molecular dynamics (followed further), the design is claimed to be successful from an in silico perspective. The baseline quality of coherent reproduction of foldings set at RMSD < 2.0 Å is an extrapolation of the traditional margin used in protein folding, protein–ligand, or protein–protein docking experiments, formally indicating the absence of significant conformational deviations and overall predictive confidence [27,28].

The scFv-0389 design pool consisted of seven total high-quality designs with RMSD_mean_ < 2.0 Å, but only two structures, namely scFv-0389-304-6 and scFv-0389-459-5, passed the individual criteria. The scFv-0743 design pool resulted in 20 total high-quality designs with only 4 passing the individual RMSD criteria, which included models scFv-0743-52-4, scFv-0743-183-2, scFv-0743-332-0, and scFv-0743-479-7. Additionally, ANARCII likelihood scores were revealed for each designed nanobody, ranging from 27.11 to 29.42, satisfying the likelihood of de novo-designed nanobodies compared to the native ones (Table 1).

### 2.4. Multistep Molecular Docking Cross-Validation

Descriptive statistics of multistep molecular docking cross-validation of RFantibody complexes shows that the scFv-0389-304-6 nanobody possess the highest Rosetta dG (dG cross) (Table 1) and interface scores (I sc) (Table 2) and its native binding pose is reproduced consistently by various tools and techniques, including either global induced-fit approach (based on a high CAPRI ranking). The scFv-0389-459-5 and scFv-0743-332-0 nanobodies can be proposed as second and third candidate nanobodies with lower binding free energies but similarly consistent docking results considering Rosetta3. It is important to state that, in terms of DockQ scoring, ClusPro2 reproduces the binding mode of nanobodies in good correlation with the Rosetta3 docking approach, at the same time allowing users to perform calculations on a free webserver and avoid a long-lasting computing routine on a local machine typical for Rosetta. Remaining nanobodies such as scFv-0743-52-4, 183-2, and 479-7 were deprecated from the further evaluation pool based on poor docking reproducibility.

**Figure 5:**
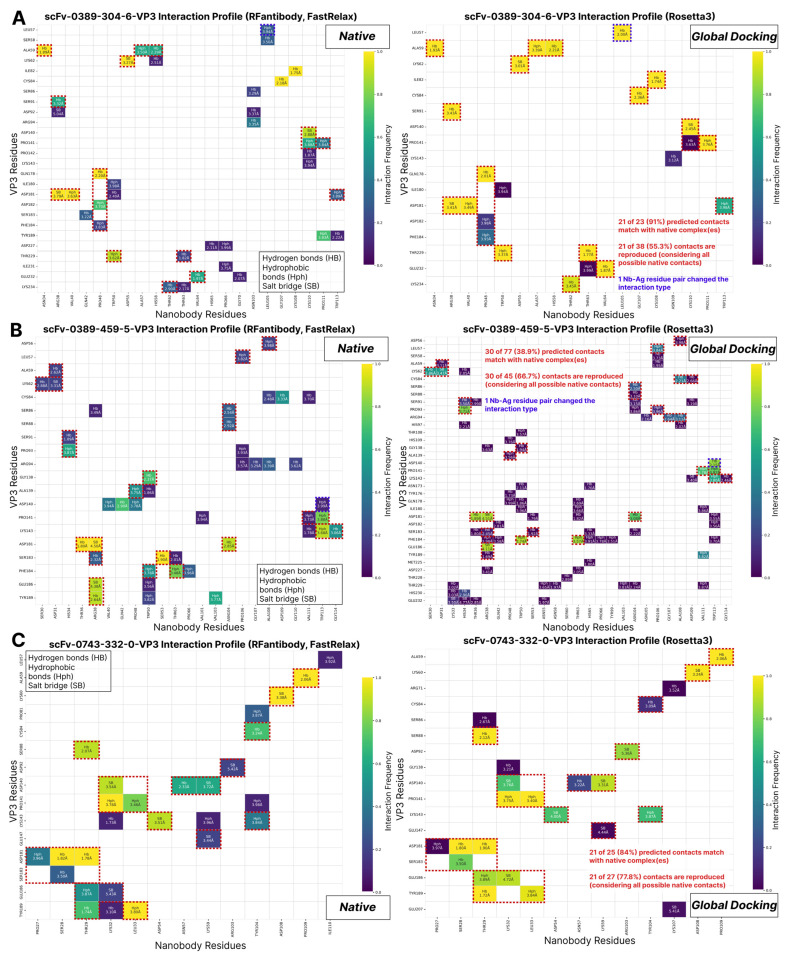
Heatmaps of interaction profiles of native (produced by RFantibody and relaxed by FastRelaxed) vs. top-100 scored (based on Rosetta interface score) complexes, predicted with Rosetta3 global docking, revealing the best reproducibility of scFv-0389-304-6 (**A**), scFv-0389-459-5 (**B**), and scFv-0743-332-0 (**C**) nanobodies binding with VP3. The VP3 structure of different complexes is renumbered according to the original 8E8Z3 sequence. Matching Nb–Ag residue pairs are depicted with red dashed lines. Interaction profiles for the native complexes were built considering 10 FastRelaxed complexes of native (produced by RFantibody) complexes.

Frequency heatmap interaction profiles were built over the top-100 complexes to visually reproduce the global docking results of the three best nanobody candidates (Figure 5). Interactions predicted with a global docking approach were compared not to a single, but to all of the FastRelaxed complexes of a given nanobody for two reasons: (1) we do not actually know the ground truth of a nanobody binding mode; (2) we aimed to capture as much as possible of the local minima variation found by Monte Carlo minimization. The brighter (from purple to yellow) the gradient on a heatmap, the higher the occurrence rate of a certain residue pair in the docking results. Furthermore, interaction types such as hydrophobic interaction (Hph), hydrogen bonds (Hbs), and salt bridges (SBs) are shown in the cells with their average distances (Å) calculated over all complexes provided below. As seen from the data, all of the complexes exhibit very consistent results when compared to RFantibody rigid body placement predictions.

### 2.5. Nanobody Humanization and Pipeline Re-Assembly

**Figure 6:**
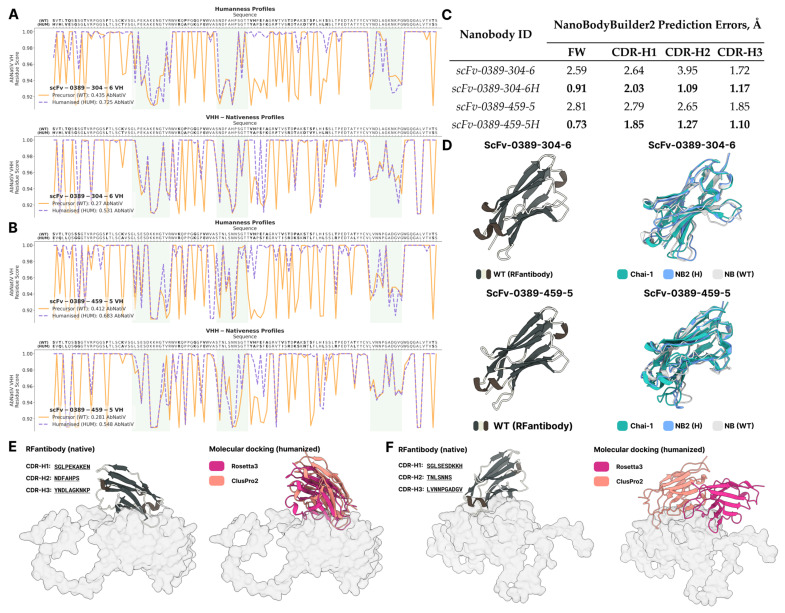
AbNatiV profiles of humanized scFv-0389-304-6 (**A**) and scFv-0389-459-5 (**B**). Table (**C**) of nanobody folding results shows structural enhancements have an almost triple to twice as low RMS score over all domains of both humanized nanobodies. (**D**) Post-CDR-design humanization resulted in relatively consistent conformations for scFv-0389-304-6H from both NB2 and Chai-1. For scFv-0389-459-5H, the nanobody humanization process resulted in higher structural mismatches in CDR regions. Relative rotations/shifts in rigid body placement of the humanized nanobodies are depicted for scFv-0389-304-6H (**E**) and scFv-0389-459-5 (**F**).

AbNatiV humanization resulted in a significant amount of mutations, increasing the VH/VHH score for scFv-0389-304-6 from 0.435/0.27 up to 0.725/0.531, and for scFv-0389-459-5 from 0.412/0.281 up to 0.683/0.548 (Figure 6A,B). Humanized sequences were re-evaluated with a modified approach to analyze the potential effects of mutations:

1. Preprocess step: Humanized sequences were folded with NB2 to estimate errors in CDR and framework regions.
2. Input complex generation: Complexes were folded with the Chai-1 [29] webserver (https://lab.chaidiscovery.com/ (accessed on 15 July 2025)) utilizing MMSeqs2 MSA and template-based modeling. At this step, we selected the Chai-1 webserver for this task, as it provides rapid, automated complex prediction based on the AlphaFold3 (AF3) architecture. Chai-1 has demonstrated near state-of-the-art performance and accuracy in protein–protein interactions prediction, particularly antibody-antigen complexes, comparable to AF3 and Boltz-1 [30], as evidenced in user-case benchmarks [31] and the Boltz-1 technical report [32]. Chai-1 also serves as a replication for RFantibody’s docking step in providing inputs for Rosetta3 global docking with post-humanized sequences, possessing a higher accuracy when compared to tFold’s models. Both web-based and local availability favored Chai-1 over Boltz-1 (which requires only local installation), though both are completely viable for that use case; Protenix was also tested for this task, but technical issues precluded its use (errors during folding).
3. Global docking (ClusPro2): Humanized nanobody structures predicted by either NB2 or Chai-1 and trimmed VP3 (used before) structure were submitted to the ClusPro2 server to screen potential binding shifts, compared to the WT complexes. Using two different nanobody models is justified at this step, because we noticed that rigid body placement in ClusPro2 is very sensitive to the input structure of a particular nanobody, and it would let us cover as many possible binding shifts in humanized nanobodies as possible. Predictions were aligned against a relaxed WT complex with the lowest dG cross to estimate the DockQ score.
4. Global docking (Rosetta3): The best complex, based on DockQ score, obtained by previous step was submitted to Rosetta3 global docking.
5. Post-docking analysis: The best complex, based on interface score, obtained by Rosetta3 global docking was vastly FastRelaxed with 50 output structures to capture more possible local minima and calculate the average dG cross. Due to the fact that the humanization of nanobodies, potentially resulting in conformational changes of CDR-loops, could affect the original binding pose by rotating the binder along the desired site, while preserving a native-like interaction profile, at this step, it would be wise to rely on the thermodynamic properties of binding rather than structural reproducibility. Nevertheless, interaction profiles were built for the set of 50 relaxed complexes and top-100 docked complexes, as well, and compared with the native (WT) complex profiles.
6. Molecular dynamics: The complex with the lowest dG cross was prepared with CHARMM-GUI and underwent 100 ns molecular dynamics simulation to compare mutation effects on nanobody flexibility, binding capability, and binding free energy.

This approach gradually increases the evaluation complexity from fast screening utilizing AF-derived models, then performs fast but a reliable global search with ClusPro and brings it together with computationally extensive Rosetta3 global docking. It is intended to allow users to control their design process and deprecate the evaluation at any step if significant dissimilarities are found.

NB2 folding error estimation resulted in a significant reduction in RMS across all CDR-loops, and the framework region, as well, for both humanized nanobodies (Figure 6C).

Chai-1 complex prediction also resulted in higher ipTM and pTM values, compared to the WT complexes (Figure 6D). Both predictions were successfully screened via ClusPro2, which resulted in the following: (1) the lowest DockQ score for scFv-0389-304-6H was the Chai-1-derived nanobody structure and was valued at 0.3789, an acceptable prediction; (2) for scFv-0389-459-459-5H, the NB2-derived structure achieved a 0.2895 DockQ score and was also an acceptable prediction. The Rosetta3 global docking predictions proved less stable, but with reproducible binding modes for scFv-0389-304-6H with half the mean interface score (compared to WT) and a mode 3 CAPRI rank calculated over 100 predictions (Figure 6E,F). ScFv-0389-459-5H nanobody top-scored complexes had a lower mode 2 CAPRI rank but a slightly higher mean interface score. Relaxed complexes retained significantly low mean dG_cross values compared to the WT complexes (Table 3). The scFv-0389-304-6H interaction profile keeps most of the original Nb–Ag residue pairs, retaining the original bonding in most of the complexes, but allowing a small fraction of non-WT-like binding poses. ScFv-0389-459-5H possesses significantly less structural similarity to its WT predecessor and very poor reproducibility (Figure S1).

### 2.6. Molecular Dynamics Simulations and Binding Free Energy Calculations

Molecular dynamics simulations of the scFv-0389-304-6/H and scFv-0389-459-5/H nanobodies confirmed low ranges of fluctuations (Figure S2A,B) and stable binding of the designed nanobodies to VP3. The humanization process affected the binding free energy for both native designs, halving its values, yet preserving strong enough and stable binding throughout the whole simulation, resulting in −37.66 ± 10.35 kcal/mol and −40.11 ± 20.01 kcal/mol for scFv-0389-304-6H and scFv-0389-459-5H, correspondingly (Figure S2C,D).

**Figure 7:**
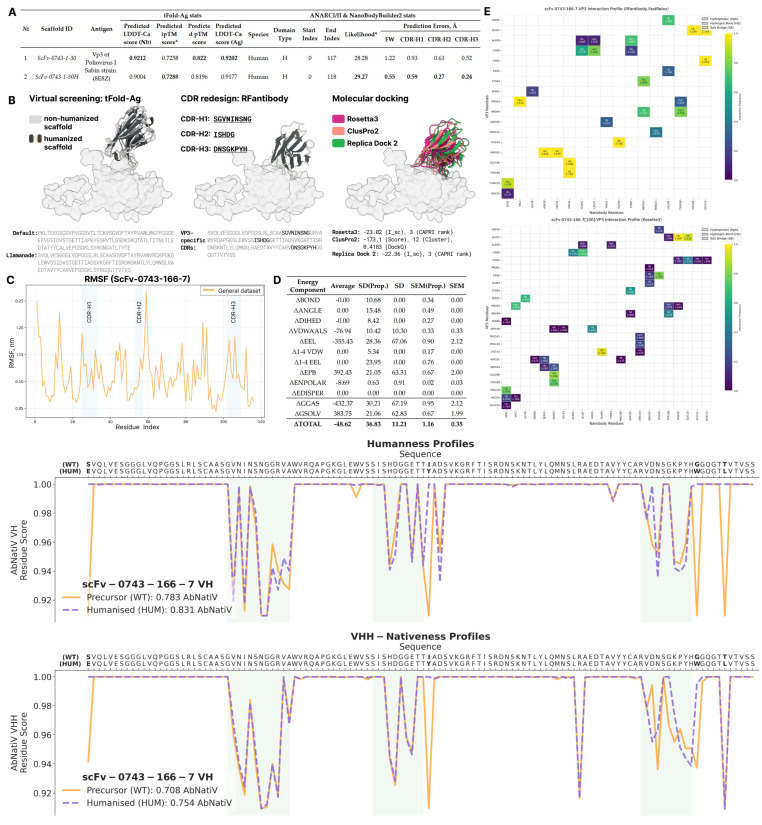
Complete graphical representation of the whole redesign pipeline utilizing the humanized scFv-0743-1-30 scaffold. (**A**) Comparison of native (ProteinMPNN) and humanized (Llamanade) scFv-0743-1-30 scaffolds shows that the latter structure gained in NB2 folding accuracy across all domains, while preserving desired tFold-Ag folding accuracy. (**B**) Graphical representation of all redesign and cross-validation steps. Utilization of humanized scaffold with RFantibody pipeline shows very consistent results in all docking cross-validation tools, required in this study. (**C**) The RMSF plot of nanobody residues through 100 ns simulation depicts a very low amplitude of fluctuations ranging from 0.5 to 2.5 Å, pointing to the high stability of the designed structure. CDR-loop regions highlighted with blue on the background possess higher mobility compared to framework regions (non-highlighted). (**D**) MM-PBSA free energy calculation (∆(Complex − Ligand − Receptor)) results performed by Uni-GBSA across 100 ns simulation (1000 frames). The data show a strong and stable bonding, valued at −48.62 ± 11.21 kcal/mol. (**E**) Interaction profile built across top-100 Rosetta3 predictions for scFv-0743-166-7 prove high reproducibility of docking, almost identically repeating the native profile. (**F**) Final humanization of scFv-0743-166-7 nanobody resulted in 4 point mutations, reaching a 0.831 VH score and 0.754 VHH score.

The scFv-0743-332-0 nanobody failed the humanization process by both Llamanade and AbNatiV tools, due to bad sequence quality (as seen before by ANARCII). Therefore, we reconsidered some design pipeline steps by humanizing the scFv-0743 framework with Llamanade (Figure 7A) and tried to graft antigen-specific CDRs onto the humanized framework, then completed all of the docking cross-validation steps, which completely failed. Then, we reattempted all of the design steps as described in the main conveyor with the humanized scaffold, starting with tFold-Ag complex pre-generation, RFantibody CDR-loop sampling, ClusPro2/Rosetta3 molecular docking cross-validation (Figure 7B), molecular dynamics structural stability assessment, and free binding energy calculation.

Of all 5000 results, only a single structure satisfied the proposed thresholds of pAE/ipAE < 4 and framework-aligned RMSD of nanobody and CDRH1-H3 < 2 Å (Table S1). Docking cross-validation of RFantibody redesign established a high level of consistency among all predictions, produced by Rosetta3, ClusPro2, and Replica Dock 2, based on either interaction profiles (Figure 7E) or docking metrics (Table S2). While the DockQ values of the best ClusPro2 docking complex may be considered below the standard of reliable docking (0.4183), we assert that even such a low value can serve as a potential validation of RFantibody results’ reliability in global docking, disregarding minor conformational discrepancies of side chains. Therefore, ClusPro2 can be used as a cross-validation method when computational resources are limited. The molecular dynamics of the scFv-0743-166-7 nanobody showed a higher level of nanobody stability, compared to the scFv-0389 designs, resulting in a lower amplitude of fluctuations ranging from <0.5 to 2.5 Å (Figure 7C). MM-PBSA binding free energy calculations (Figure 7D) revealed energies of −48.62 ± 11.21, which were lower than those of the scFv-0389 designs throughout the simulations. When performing AbNatiV humanization for the native sequence, we found that the originally produced sequence was insufficiently long (145 amino acids) when it was renumbered according to the AHo scheme used by the tool. Inspection of the humanized scFv-0743-1-30 scaffold revealed that the TVS fragment at the end of the RFantibody design was missing. Therefore, we manually appended the TVSS fragment to the end of the design, reaching the required length of 149 amino acids (the terminal serine (S) residue was appended due to its dominant presence in most of the nanobodies (see also Figure 3F)). Four point mutations were proposed, which increased the VH and VHH scores from 0.783 and 0.708 to 0.831 and 0.754, respectively (Figure 7F).

### 2.7. Physicochemical Evaluation of Nanobodies

Final evaluation of the designed nanobodies in terms of physicochemical and allergenicity parameters exhibited the following results: (1) All nanobodies possess good values of melting temperature ranging from 62.34 °C to 63.69 °C. (2) Humanization of all nanobodies significantly improved solubility and allergenicity scores (except for scFv-0389-304-6H) (Table 4, Figure S3). (3) The predicted isoelectric points (pI) are within a 7.80–8.68 range; all nanobodies are claimed to be stable based on the Stability Index, ranging from 29.13 to 38.67; and the estimated time of half-life ranges from 1 to 3.5 h, which is considered to be representative for most of the nanobodies (Table S3) [9]. Multiple sequence alignment (MUSCLE) and phylogenetic tree building (IQ-TREE) via UniproUGENE comparing final sequences and previously utilized crystal structures of nanobodies, bound to VP3 demonstrated distinct clusters of scFv-0389 designs, the scFv-0743-166-7/H design, and in vitro derived nanobodies, among which scFv-0743-166-7/H was the most cognate to experimental designs (Figure S4). Of all designs, scFv-0743-166-7/H nanobodies possess the highest level of similarity with real structures—reaching ≥60% identity (Figure S5).

## 3. Discussion

In this study, we have successfully designed new Poliovirus I Sabin strain-specific nanobodies and shared our design pathway, as well as the full bioinformatic pipeline, with the scientific community. This pathway encompasses the most recent advances in nanobody/antibody and protein modeling tools and methods. From the perspectives of virology and microbiology, our computational approach has the potential to benefit researchers by reducing the cost and duration of nanobody production. This is highly relevant given the rapid evolution of new pathogenic viruses [33] or microorganisms [34].

De novo nanobody design remains a computational challenge as mentioned by Bennet et al. [35]. New AI-driven methods, such as new protein folding techniques (AlphaFold3, Chai-1, etc.), or various DL-models (Ig-VAE, DLAB [36], AntyBERTy [37], NanoBERT [38], IgLM [39], RefineGNN [40], Chai-2 (https://www.chaidiscovery.com/news/introducing-chai-2, (accessed on 25 July 2025) etc.) capable of either helping at some stages of nanobody design or producing ready-to-use antibodies with high success rates are gaining significant popularity due to their cost-effectiveness and lower time and effort demands, though they remain speculative—as traditional in vitro library preparation and in silico homology modeling methods remain valid and reliable—as well as being unavailable to a broad variety of researchers. Furthermore, the current problem persisting in the scope of antibody/nanobody design is that, on the one hand, new computational methods and tools are addressing nanobody design problems only at distinct steps (for instance, only folding or only scaffold sampling), but on the other hand, are encompassing the whole design process, fully relying on language models and protein embedding. As previously mentioned, producing nanobodies can be costly using traditional techniques and heavily relies on previous successful experimental designs for certain antigens in the case of homology modeling [41]. Therefore, the primary objective was to maintain all of the benefits of computational nanobody design by establishing an integrated pipeline of deep learning tools and conventional molecular docking and ab initio methods. This approach enables the tracking of all design steps, manual adjustment of experiment settings, and the elimination of the requirement for a valid VH framework at the start.

Firstly, to identify the most suitable target(s) of Poliovirus capsid proteins we explored existing crystal complexes of in vitro-obtained antibodies and nanobodies (PDB-ID: 3JBC, 3JBD, 3JBE, 3JBF, 5KTZ, 5KU0, 5KU2, 5KWL). Analysis showed that Virus Protein 3 was predominantly targeted by all nanobodies, at the Asp56, Leu57, Ser58, Ala59, Lys60, Lys62, Val70, Arg71, Pro81, Ile82, Leu83, Cys84, Ser91, Asp92, Pro93, Pro141, Pro142, Lys143, Ile180, and Asp181 hotspots. These residues cover the surface of VP3 that is most adjacent to VP1 and VP2 subunits, serving as a valid orientation for blocking the D-antigen area [42].

Addressing the main challenge, we vastly sampled 1000 nanobody scaffolds by Ig-VAE, then based on the obtained structures found the best possible sequence candidates by ProteinMPNN (three top-score sequences per scaffold). Multiple filtration procedures were executed to let only the best designs pass, relying on a deep learning approach (tFold-Ab, tFold-Ag), which was significantly time-effective compared to traditional docking virtual screening experiments. At the point of obtaining top-score candidate framework scaffolds, our pipeline was split into two distinctive ways to gain the perfect design, as was shown later in the Section 2: (1) perform the antigen-specific design of CDR-loops, evaluate them using a global and induced-fit docking approach, humanize successful nanobody sequences, and recurrently re-evaluate humanized sequences; (2) perform the humanization of top-score scaffolds, then sample antigen-specific CDR-loops and approve the binding capability via the same docking approach. Such variations were considered at the beginning of our study but needed real-case validation. We decided to share the whole process, including failed designs and possible obstacles that otherwise could certainly be met by anyone following our steps. The recently available open-source RFantibody tool had a key role in our conveyor, the results of which we have proven to be effective and reliable, validating them by three absolutely divergent tools, including Rosetta3 global docking, ClusPro2 global docking, and Replica Dock 2 induced-fit docking. We showed that our own determination of successful RFantibody CDR-designs based on strict pAE/ipAE and RMSD clustering resulted in higher reproducibility for sequences derived by the v_48_020 ProteinMPNN model. The sequences of the v_48_030 model failed using sequence-based prediction and estimation tools, such as NanoBodyBuilder2, ANARCI/II, or AbNatiV. In order to save the time and effort spent on modeling the v_48_030 dataset, we arbitrarily humanized the scFv-0743-1-30 scaffold which appeared to be even higher quality in almost all later experiments than the original approach. Only one structure fitted the desired pAE/ipAE and RMSD clustering, namely scFv-0743-166-7. All three molecular docking tools validated the RFantibody predictions and proved the reproducibility of the nanobody complex. In order to validate our designs by non-experimental methods, we finalized the study with molecular dynamics simulations of native and humanized complexes to explore the effects of humanizations and overall nanobody structural stability, as well as to generate input trajectories for MM-PBSA binding free energy calculations. All models possessed the expected low range fluctuations in framework regions and higher values in CDR regions. The binding free energy of all complexes had stable values with mid-level to low fluctuations. In terms of physicochemical properties, which are arbitrary to follow and serve as additional validation methods, all humanized nanobodies possess acceptable values of solubility and melting temperature. Allergenicity predictions determined all nanobodies as non-allergenic, except for scFv-0389-304-6. Nevertheless, we successfully observed acceptable mutations to eliminate allergenicity profiles in framework regions and enhance solubility, if required.

Comparing the results of humanized nanobodies with predesigned CDRs (scFv-0389H designs) and nanobody with post-humanization CDR design (scFv-0743-166-7/H), the latter resulted in higher reproducibility and stability values in docking experiments, preserving a high humanness score, lower fluctuations and binding free energy values in molecular dynamics simulations, a low allergenicity score, and even perfect solubility and half-life time values. Even more, the latter design retains a higher identity index and closer phylogenetic likelihood distance compared to real nanobodies specific to VP3.

**Figure 8:**
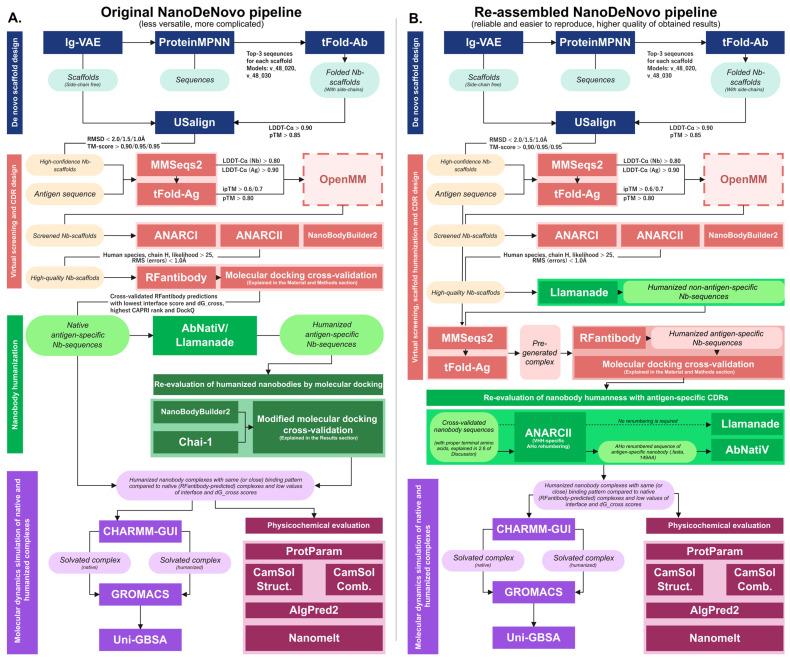
(**A**) Original approach of de novo nanobody design conveyor, composed of 5 consecutive stages consisting of (1) nanobody scaffold structure and sequence sampling; (2) scaffold–antigen (Nb-Ag) complex virtual screening, optimization, filtering, and CDR design with further multistep molecular docking cross-validation; (3) humanization of the best nanobody sequences with binding properties re-evaluation; (4) molecular dynamics simulation of native and humanized complexes for structural stability assessment with further MM-PBSA binding free energy calculations; (5) assessment of de novo-designed nanobodies by open-source web services to predict their physicochemical and allergenicity properties. (**B**) The computationally validated (scFv-0743-166-7/H) production version of the NanoDeNovo pipeline, which includes nanobody scaffold humanization prior to CDR design, yields better results in terms of docking cross-validation, ab initio/free energy calculations, physicochemical properties, and the overall integrity of the utilized tools and intermediate results obtained, decreasing the complexity and number of steps required to obtain the best possible nanobody design.

Based on practical considerations (tools workflow and functionality) followed by computational validation, proving coherence between the tools and the obtained results, we state that the CDR sequence design for non-humanized nanobody sequences is considered less versatile than post-humanization CDR design. The latter version of the conveyor possesses a higher successful design capability and better integration of several tools, allowing researchers to exclude additional validation steps, with the main choice being to require extra folding and extra docking cross-validation steps. The overall workflow of the original (Figure 8A) and re-assembled (Figure 8B) antigen-specific nanobody sequence and structure design pipeline with ab initio evaluation including physicochemical properties prediction of best nanobody structures and molecular dynamics simulations of post-docked complexes is depicted in Figure 8.

While our computational pipeline demonstrates promising designs for Poliovirus I Sabin strain-specific nanobodies, it is inherently limited as a purely in silico study. Key limitations include the reliance on predictive models whose accuracy depends on training datasets, which may not fully represent the structural variability of viral capsids in biological environments. For instance, molecular dynamics simulations and MM-PBSA calculations provide insights into stability and binding free energy but cannot account for experimental artifacts like aggregation, off-target effects, or immune responses. Docking tools (e.g., Rosetta3, ClusPro2, Replica Dock 2) validated our predictions, but these are approximations that may overestimate affinities without wet-lab confirmation. Additionally, physicochemical predictions (e.g., solubility, allergenicity) are based on algorithms with known false positives/negatives, as seen in the allergenicity flag for scFv-0389-304-6. These constraints underscore that our results are preliminary and require empirical validation to confirm therapeutic potential.

In summary, our computational pipeline integrated AI-driven scaffold and sequence sampling (Ig-VAE, ProteinMPNN), CDR design (RFantibody), docking validations (Rosetta3, ClusPro2, Replica Dock 2), simulations (molecular dynamics, MM-PBSA), and nanobody humanization tools (AbNatiV, Llamanade) to generate humanized nanobodies targeting Poliovirus VP3. Key findings include the superior reproducibility of post-humanization CDR design (e.g., scFv-0743-166-7/H), with stable binding energies, low allergenicity, and favorable physicochemical properties. Phylogenetic analysis further supports its similarity to known VP3-specific nanobodies. However, these results are derived solely from in silico methods and must be interpreted cautiously. The essential next step is experimental validation, including in vitro expression, binding affinity tests (e.g., ELISA or SPR), structural confirmation (e.g., cryo-EM), and in vivo efficacy studies in animal models, to bridge the gap between computational predictions and practical applications in drug discovery.

## 4. Materials and Methods

### 4.1. Antigen Structure Preparation

In the present study we took the Poliovirus I Sabin strain Virus Protein 1-4 complex (VP1-VP4) crystal structure from RCSB-PDB (https://www.rcsb.org (accessed on 10 February 2025)) assembly 8E8Z, 9H2 Fab-fragment, and the VP1-2 and VP4 capsid proteins were deleted (https://doi.org/10.2210/pdb8E8Z/pdb, (accessed on 10 February 2025)). The structure was prepared by PDFixer [43], omitting water molecules and non-antigen heteroatoms, and adding polar hydrogens according to the blood pH level, equal to 7.4.

### 4.2. Nanobody Structure and Sequence Design and Optimization

One-thousand nanobody protein scaffolds composed only of C, N, O, and Cα-atoms, representing nanobody backbones without side-chain information, were generated via the Ig-VAE autoencoder [44]. Further, for each of the sampled structures, 128 amino acid sequences were designed by ProteinMPNN [45]. As the input parameters, we empirically determined that the v_48_020 and v_48_030 weights with temperature sampling (T) of 0.15 had the most optimal sampling results, having enough sequence recovery in variable regions (CDR-H3 loop) and remaining relatively constant in the framework region. For each model weight group, 3 top-score sequences were extracted, resulting in 6000 nanobody sequences total (3000 for each of the weight models). Final nanobody structure generation was performed by the tFold-Ab model [46,47] in an MSA-free manner; for dataset optimization and structure quality filtering purposes, each predicted nanobody structure was sorted by LDDT-Cα (Local Distance Difference Test scoring of Cα-atoms) > 0.90 and pTM (predicted Template Modeling score) > 0.85 (provided within the pdb file of a model); then, a refined dataset of high-confidence structures was aligned via USalign [48] based on Cα-atoms with its corresponding Ig-VAE scaffold dataset, where a designed nanobody was aligned against its sidechain-free scaffold. Those with RMSD (Root Mean Square Deviation) > 2.0 Å and TM-score < 0.90 were deprecated from the design pool. Plots and statistics calculations were performed by custom code written in the Python3 programming language utilizing the matplotlib (ver. 3.10.1), numpy (ver. 2.2.4), and pandas (2.2.3) packages.

### 4.3. Nanobody–Antigen Complex Virtual Screening via Deep Learning Approach

The first approach of a rigid body placement in a virtual screening manner of designed nanobodies along the antigen structure was performed via the tFold-Ag model [49]. The process started with generating the .a3m MSA-files for the VP3 protein sequence (obtained from 8E8Z) as the model was not yet optimized for precise multi-domain antigen structure prediction, giving the error when overcoming the predetermined amount of chains in a multi-fasta file (1, 2, or 3) in the case of the whole VP–complex structure prediction. Also, all of the available crystal structures of nanobodies bound to the Poliovirus I target VP3, specifically the part on the border of the VP1–VP2–VP3 complex, called the D-antigen [50]. Multiple alignment was performed by tFold-Ag’s gen_msa.py module with preinstalled MMSeqs2 software [51] and locally installed uniref30, colabfold, and pdb100 databases, following the tFold installation guide. For the proper functionality of the gen_msa.py paths, these databases were adjusted according to their installation directories, as well as the maximum number of threads available on the local CPU-cluster (38 cores/76 threads, 512GB RAM) for performance purposes, and the MMSeq2 installation directory. Then, having the .a3m MSA-file of the VP3 protein, we performed the folding process by executing the predict.py module, utilizing concatenated fasta files filtered at the second step of nanobody sequences and the Poliovirus I Sabin strain VP3 sequence with the corresponding .a3m MSA-file of VP3.

After Nb–VP3 complexes were generated, we performed another data processing step aiming to pick only mid- to high-confidence complexes based on several AlphaFold-derived prediction score metrics. The bottom threshold was LDDT-Cα of nanobody >0.80, LDDT-Cα of antigen (VP3) > 0.90, pTM > 0.80, and ipTM (interface-predicted Template Modeling score) > 0.7. The key limiting parameter during this filtration process was the ipTM score, depicting how confident we are in the structure of the predicted interacting residues (in our case, the VP3 D-antigen pocket and CDR-H3 loops of designed nanobodies) in our nanobody–antigen complex pool, and according to the EMBL-EBI AlphaFold tutorial we set the ipTM value threshold at a minimum of 0.7 (a little higher than the default “grey zone”), thus requiring additional validation.

All of the complexes that passed this threshold were minimized using OpenMM 8.2 package [52] utilizing the Amber14SB force field [53] due to structural clashes and unrealistic interatomic distances found across all structures during the tFold prediction process. The maximum level of iterations was set at 1000 (+100 step size) with termination at 10 kJ/mol threshold of energy change as the point of reaching convergence.

Further analysis of the virtual screening step was undertaken to evaluate all of the designed nanobodies utilizing two extra tools—ANARCI and ANARCII antibody domain identification [54,55]—proving our designed nanobody sequences are H-chains indeed; to obtain the confidence levels of the designed sequences; to investigate the presence of missing sequence information in variable regions; and to use NanoBodyBuilder2 as a built-in package of ImmuneBuilder (https://github.com/oxpig/ImmuneBuilder (accessed on 10 March 2025) [56], as an additional assessment of tFold-Ab/Ag models also capable of identifying the CDR-loop structure prediction accuracy (in terms of prediction errors, described in the original paper).

### 4.4. CDR-H Loop Design by RFantibody Pipeline

After the complete virtual screening of potential nanobody candidates, we performed the additional CDR-H loop design by the recently released RosettaFold Antibody (RFantibody) pipeline (Figure S6A) (https://github.com/RosettaCommons/RFantibody (accessed on 15 March 2025)) [57]. The top-rated nanobody structures, one from each of a model weight group, were used as complete framework structures, and the VP3 antigen was used as the target. Before the sampling process, we identified the hotspot regions of the VP3 by summarizing all of the residues potentially required for successful binding of the nanobody in the D-antigen region, based on successfully validated virtual screened complexes and available crystal structures using PLIP software (PharmAI GmbH, Germany) [58]. The hotspots included the following residues: Asp56, Leu57, Ser58, Ala59, Lys60, Lys62, Val70, Arg71, Pro81, Ile82, Leu83, Cys84, Ser91, Asp92, Pro93, Pro141, Pro142, Lys143, Ile180, Asp181. Additional proprietary HLT-formatting of PDB files was performed for the minimized complexes as a whole, then split into VP3 proteins as targets and nanobodies as frameworks. CDR-loops were manually adjusted in HLT-formatted pdbs, according to the Chothia definition obtained from the NB2 validation step. The whole process ensures proper atom renumbering, as processing standalone VP3 as a target followed by RFdiffusion inference sampling on the first step caused structural artifacts.

The sampling parameters of the rfdiffusion inference step adjusted in the present study are as follows: ‘ppi.hotspot_res = [T56,T57,T58,T59,T60,T62,T70,T71,T81,T82,T83,T84,T91,T92,T93,T141,T142,T143,T180,T181]’, ‘antibody.design loops = [H1:10,H2:7,H3:10]’, inference.num designs = 500 (number of docked complexes). The others remained unchanged. The CDR3 loop length was set to 10 amino acids, the default value provided in the original software instructions, to ensure a reasonable sampling time on the GPU. This value may not be biologically reasonable. The proteinmpnn inference step parameters were set as follows: loop string, H1, H2, H3; seqs per struct, 10; num connections, 96 (multiplied by 2 from the default for precision purposes). Parameters of the rf2_inference step of fine-tuned RFdiffusion inference were kept as default. The overall process resulted in 5000 designs (half that recommended by default for performance purposes).

Data analysis of the best structures obtained was performed by qvscore script, modified by software, enabling csv and xlsx table generation with design stats (see Data Availability Statement Section). As recommended in the original RFantibody repo, we filtered out the structures with the lowest possible combination of ipAE (interaction-predicted aligned error score); pAE (predicted aligned error score) < 4.0; set of framework aligned CDR, H1-3 loop root mean squared deviation scores < 2.0 Å; and visual assessment of desired nanobody orientation along the VP3 structure.

The evaluation of RFantibody design was supported with Rosetta structure refinement (FastRelaxed) with calculation of the dG cross binding score of the obtained complexes (see Data Availability Statement Section). Refinement was conducted 10 times (-nstruct 10) for each complex and score values were calculated as averages. The conformational state of the filtered nanobody with redesigned CDR-loops and cross-validated binding pose by the molecular docking method (described further) with the lowest dG cross score served as the input structure for molecular dynamics simulation and physicochemical properties analysis.

### 4.5. Molecular Docking and Structural Cross-Validation of Designed Nanobodies

Molecular docking of non-energy refined Nb–Ag complexes was performed with different Rosetta3 Docking [59] and ReplicaDock 2.0 (RD2) [60] docking protocols—hotspot-free global docking and directed induced-fit local docking (Figure S6B)—also utilizing the ClusPro2 (CP2) docking server (https://cluspro.bu.edu/home.php (accessed on 18 May 2025).

For each of the filtered designed complexes, we performed 25 repacking attempts (for nanobody and antigen structures separately), excluding the antigen’s hotspots, to prevent any possible structural clashes, then sorted out structures with the lowest Rosetta SCORE value and concatenated them into a single complex file. The first 40 amino acid residues of the VP3 protein were trimmed in production runs to prevent the rigid body placement step from trapping nanobodies in the β-loop caveat, revealed in test runs (Figure S7A), which is natively hidden from the hotspot surface by the VP1 subunit in the whole Poliovirus VP-complex (Figure S7B).

For Rosetta3 and ClusPro2 protein–protein docking approaches, we conducted a global search of possible conformational states (10,000 conformations for the Rosetta3, and 70,000 for ClusPro2, as the default) of the designed nanobodies to confirm the reliability of RFantibody predictions. The Rosetta3 nanobody CDR-loop residues were defined in the RESIDUE_SELECTORS block, and the protocol consisted of a relaxation step (FastRelaxed mover), low-resolution centroid docking (Docking mover with fullatom = “0”), and high-resolution full-atom docking (Docking mover with fullatom = “1”). For ClusPro2, we split the repacked complexes into nanobody and antigen parts, enabling Antibody Mode, where the nanobody was stated as a receptor and the antigen as a ligand, then launched an FFT-based rigid docking search.

The directed induced-fit local docking protocol was implemented to refine pre-aligned nanobody–antigen complexes, emphasizing optimization of the binding interface while accommodating backbone flexibility to capture induced-fit conformational changes. This procedure utilized Rosetta’s docking framework, guided by the XML parameter file replicadock.xml (see Data Availability Statement Section). The docking simulation employed two score functions: the low-resolution muds2021 score function (score dock low) for sampling and the motif-based motif dock score (score analyze) for post-docking analysis. Rigid-body transformation (docking jump) was established by DockSetupMover, and RigidBodyPerturbNoCenter introduced small rigid-body perturbations (1° rotation, 1 Å translation). Backbone flexibility was incorporated via the Backrub mover, applied to previously determined residue ranges (CDR-loops for nanobodies and hotspots for VP3) with a slope of −0.5. Enhanced sampling was achieved using HamiltonianExchange for parallel tempering, with temperature schedules specified in a separate file provided by default. Additional movers, such as DockingInitialPerturbation, TrialCounterObserver, and SilentTrajectoryRecorder, initialized the pose, tracked trials, and recorded simulation trajectories, respectively. The MetropolisHastings mover executed a Monte Carlo simulation with 1,000,000 trials per replica, integrating the above movers with a sampling weight of 3 for backbone movements.

For the evaluation of docking results in Rosetta3, the DockingMetrics option was set true in the option file. The ReplicaDock 2.0 protocol required an additional refinement process added to the run .sh pipeline storing only score files, to extract a wider range of docking statistics, including SCORE, total score, CAPRI rank, I sc, rms, etc., into a high_res.sc score file. For RMS computing, repacked complexes were set as natives for both docking procedures under the in:file:native flag. Based on I sc scoring, the top 100 for global search and top 5 for directed induced-fit docking complexes were extracted from scores.sc and high_res.sc, correspondingly.

Analysis of docking complexes for all of the attempted approaches was performed by CAPRI-Q on the Dockground webserver (https://dockground.compbio.ku.edu/assessment/ (accessed on 20 June 2025), evaluating the DockQ score and classification of docked complexes. The DockQ metric references three basic CAPRI metrics for protein–protein docking [61], including Fnat (fracture of native contacts), lRMSD (nanobody RMSD with fitted antigen), and iRMSDbb (RMSD of interfacing residues backbones), serving as a reliable representation of molecular docking quality. The heatmaps were built for each dataset of top-rated docked complexes using interactions revealed by the PLIP package with the --peptides and --nohydro modes set.

### 4.6. Molecular Dynamics of Nanobody–Antigen Complexes

Molecular dynamics simulations were conducted for the relaxed (FastRelaxed) complexes that passed the molecular docking cross-validation step via GROMACS software (ver. 2023.3,CUDA, single precision) [62]. Solvation boxes were prepared with the CHARMM-GUI webserver (https://www.charmm-gui.org/ (accessed on 18 July 2025) [63]. Protonation state was set at 7.4 (as blood native), and possible disulfide bond(s) recognition was enabled. Then the system was solvated by the periodic boundary conditions method in a cubic region with a minimum distance of boundary atoms of 10 Å, and K^+^ and Cl^−^ ions were added to the system at 0.15 mM concentration by the Monte Carlo method in order to neutralize the system. Finally, the system was parameterized with an AMBER force field, utilizing ff19SB [55] for the proteins and OPC as a water model.

From the first step, the existing index file was updated by adding custom groups—nanobody (LIGAND), VP3 (RECEPTOR), solvent (SOLV), and protein–protein complex (LIGAND_RECEPTOR)—required for further energy calculations. The prepared complexes were minimized using the steepest descent method with 1000 kJ/mol/nm set as the point of reaching convergence. Multiple steps were executed to equilibrate the system: First, the NVT-ensemble was simulated utilizing a Nose–Hoover thermostat for 1 ns to equilibrate the system temperature at 303.15 K with two temperature baths specified for the protein–protein complex and the solvent, then three consecutive NPT-ensembles were simulated by a C-rescale barostat for 100 ps (restraining all bonds), 100 ps (restraining H-bonds), and 2 ns (restraining all bonds) with the reference pressure set at 1 bar. Equilibrated complexes were used for 100 ns production simulations, using the leapfrog method; temperature and pressure coupling were performed with a Parrinello–Rahman thermostat and a Nose–Hoover barostat. For all steps, LINCS was used as a constraint algorithm, short-range electrostatic and van der Waals (VdW) cut offs were set at 10 Å and 8 Å, correspondingly, calculated with AMBER-specific Potential-shift-Verlet modifier, and long-range electrostatics were calculated with the Particle Mesh Ewald method.

All of the production topologies were re-centered to prevent visual artifacts, and then submitted to the MM/GB(PB)SA free energy calculation step. RMSF plots for nanobody residues (calculated as averages) were built to assess possible structural fluctuations in the designed nanobodies.

### 4.7. MM/GBSA and MM/PBSA Binding Energy Estimation

Binding free energy calculations were performed via the Uni-GBSA tool (https://github.com/dptech-corp/Uni-GBSA (accessed on 21 July 2025)) [64] (unigbsa-traj) utilizing the gmx_MMPBSA framework [65] by both the Generalized Born Surface Area (GBSA) and Poisson–Boltzmann Surface Area (PBSA) methods across entire production simulations. The AMBER ff19SB force field was defined to build topology, temperature was set at 303.15 K, and salt concentration was set at 0.15 M; the remaining parameters were set to the default as generated by the tool.

### 4.8. Nanobody Humanization

The best sequences that passed all of the in silico design and validation steps were scored and humanized with the LLamanade webserver (http://www.llamanade.app/ (accessed on 10 July 2025)) [66] and AbNatiV software (https://gitlab.developers.cam.ac.uk/ch/sormanni/abnativ (accessed on 10 July 2025)) [67].

### 4.9. Physicochemical Properties Prediction

Nanobodies were evaluated by their melting temperatures via NanoMelt (gitlab.developers.cam.ac.uk/ch/sormanni/nanomelt (accessed on 22 July 2025)) [68] software.

Inheriting the best practices of computational nanobody design from Poustforoosh et al. [69], we attempted to assess the structural and physicochemical properties of best nanobody designs via ProtParam (https://web.expasy.org/protparam/ (accessed on 22 July 2025)) [70]. The solubility of the nanobodies was assessed by the CamSol Structurally Corrected tool (https://www-cohsoftware.ch.cam.ac.uk/index.php/camsolstrucorr (accessed on 22 July 2025)) [71,72] utilizing NB2-derived structures cleaned on the same server with pH = 7 and PatchRadius = 10 Å for all designs. A potential search of point mutations for solubility enhancement was performed with the CamSol Combination method (https://www-cohsoftware.ch.cam.ac.uk/index.php/camsolcombination (accessed on 22 July 2025)) for all humanized nanobodies with the following parameters:

1. Antibody/nanobody mode—yes;
2. Alignment frequency strong filter—yes;
3. Use frequency PSSM (PWM)—yes;
4. Exclude these potential substitution target residues—M, C, N;
5. Residues that cannot be changed—proprietary for each nanobody;
6. Automated Chain Similarity Check—yes;
7. Maximum Simultaneous Mutations in Combinations—8.

Allergenicity predictions were performed using AlgPred2 (https://webs.iiitd.edu.in/raghava/algpred2/ (accessed on 22 July 2025)) [73] with the AAC-based RF method, both with a threshold of 0.4. Potential single-point mutations to enhance the non-allergenic properties of nanobodies were searched for on the same server for all successful humanized sequences. We then re-evaluated the proposed mutations for the sequences with the lowest non-allergenicity score.

The enhancements proposed by the AlgPred2 and CamSol combination tools are arbitrary and are provided to accomplish the overall design process, especially when considering all possible factors before potential in vitro experiments.

### 4.10. Visualization

All molecular visualizations were performed with ChimeraX v.1.10 (UCSF RBVI, San Francisco, CA, USA) [74].

## 5. Conclusions

In this paper, we assembled a reproducible semi-automated program pipeline for the de novo design of antigen-specific nanobodies with high target specificity. The pipeline was utilized to design nanobodies specific to Virus Protein 3 of the Poliovirus I Sabin strain. Four validated humanized nanobodies were obtained: scFv-0389-304-6H, scFv-0389-459-5H, and scFv-0743-166-7/H.

## Figures and Tables

**Table 1:** RFantibody design statistics.

Scaffold	Design IDs	ipAE	pAE	Framework-Aligned RMSD of, Å					Mean dG_cross ^1^	ANARCII Score
				Nanobody	CDRs	H1	H2	H3		
ScFv-0389	304-6	2.29	3.12	0.9	1.29	1.08	0.61	1.76	−57.93	29.19
	459-5	2.44	3.13	1.03	1.4	1.35	1.15	1.6	−48.68	29.42
ScFv-0743	52-4	1.84	2.95	1.04	1.67	1.55	1.61	1.82	−41.86	27.20
	183-2	2.12	3.31	1.04	1.68	1.14	1.84	1.98	−33.07	27.11
	332-0	2.07	3.47	1.03	1.61	1.83	1.33	1.54	−51.75	28.11 ^2^
	479-7	2.33	3.91	0.8	1.03	0.7	1.24	1.13	−46.35	27.21

^1^ Calculated over 10 FastRelaxed complexes for each scaffold. ^2^ The sequence is heavily truncated when renumbered.

**Table 2:** Molecular docking cross-validation results and CAPRI-Q evaluation.

Nanobody/ Scaffold	Number of Total Complexes/Clusters		Mean Interface Score for Rosetta3 ^1^	Rosetta3 CAPRI Rank Mode ^1^	Mean Interface Score for RD2	RD2 CAPRI Rank Mode ^1^	Lowest CP2 score ^2^/ Cluster Rank	Highest CP2 DockQ Score ^2^ (Classification)
	Rosetta3	CP2						
ScFv-0389-304-6	8079 (80.79%)	30	−56.02	2	−47.03	3	−227.0/13	0.6406 (medium)
ScFv-0389-459-5	7351 (73.51%)	30	−20.43	2	−29.66	3	−283.2/1	0.6792 (medium)
ScFv-0743-52-4	6915 (69.15%)	30	−17.53	2	−21.37	2	−256.0/5	0.3894 (acceptable)
ScFv-0743-183-2	7312 (73.12%)	30	−15.85	1	−17.71	0	No match	N/A
ScFv-0743-332-0	7737 (77.37%)	24	−31.93	3	−27.07	2	−237.1/9	0.3438 (acceptable)
ScFv-0743-479-7	7659 (76.59%)	30	−20.73	2	−17.84	0	−203.5/18	0.2607 (acceptable)

^1^ Calculated over top-100 complexes, ranked by I sc. ^2^ Taken for the complex with the best DockQ compared to the native complex.

**Table 3:** Repetitive assessment of humanized nanobodies.

Nanobody/ Scaffold	Number of Total Complexes/Clusters		Mean Interface Score for Rosetta3 ^1^	Rosetta3 CAPRI Rank Mode ^1^	Mean dG Cross ^3^	Lowest CP2 Score ^2^/ Cluster Rank	Highest CP2 DockQ Score/Folder ^2^ (Classification)
	Rosetta3	CP2					
ScFv-0389-304-6H	7396	30	−25.50	3	−55.18	−239.6/0	0.3789/Chai-1 (acceptable)
ScFv-0389-459-5H	7606	26	−21.6	2	−52.82	−184.1/28	0.2895/NB2 (acceptable)

^1^ Calculated over top-100 complexes, ranked by I sc; in this particular case, an input complex served as a reference for CAPRI calculation. ^2^ Taken for the complex with the best DockQ compared to the FastRelaxed complex; for humanized nanobodies we picked the best score possible considering both NB2 and Chai-1 structures. ^3^ Calculated over 50 FastRelaxed complexes.

**Table 4:** Complete characteristics of VP3-specific de novo-designed nanobodies.

Nanobody	Sequence ^1^	Solubility Estimation		Allergenicity Estimation		Nativeness			T_m_, °C
		CamSol Struct. Score ^2^	CamSol Comb. Mutations (Single Letter) ^3^	AlgPred2 Score ^4^	AlgPred2 Mutations (Single Letter) ^5^	VH-ness	VHH-ness	Llamanade	NanoMelt
VH-0389-304-6	SVTLTQSSSGTVRPGGSFTLSCKVSGLPEKAKENGTVRWVKQPPGGGPVWVASNDFAHPSGTTVHPEFAGRVTVSTDPAKSTSFLHISSLTPEDTATYYCVYNDLAGKNKPGWGQGALVTVT**S** (Serine (S) was appended as a terminal residue to prevent side-chain packing error on the NB2 webserver. Originally, proline (P) persisted in the scFv-0389 framework, but was trimmed by RFantibody. During MSA comparison, we manually substituted proline on serine, as it appears in almost all nanobodies (also see Figure 3F).)	0.29/1.85	N/A	0.431	N/A	0.435	0.27	56.8	63.44
VH-0389-304-6H	HVHLVESGSGLVRPGGSLTLSCTVSGLPEKAKENGTVRWVRQAPGKGPEWVASNDFAHPSGTTYAPSFKGRFTVSRDTAKDTVYLHLNSLTPEDTATYYCVYNDLAGKNKPGWGQGALVTVSS	0.66/1.91	H1E, H3Q, T23K, T63R, A79S	0.464/0.367	N34R, N54R, D55, D77R, D81R, N88R, D94R, N103R, D104R, N109R	0.725	0.531	74.29	63.23
VH-0389-459-5	SVTLTQSSSGTVRPGGSFTLSCKVSGLSESDKKHGTVRWVKQPPGGGPVWVASTNLSNNSGTTVHPEFAGRVTVSTDPAKSTSFLHISSLTPEDTATYYCVLVNNPGADGVGWGQGALVTVTS	−0.02/1.74	N/A	0.382	N/A	0.412	0.281	56.6	63.69
VH-0389-459-5H	EVQLLQSGGGTVRPGGSLTLSCAVSGLSESDKKHGTVRWVRQPPGKGPEWVASTNLSNNSGTTYAPSFEGRVTISRDKSKNTLFLHLSSLRPEDTALYYCVLVNNPGADGVGWGQGALVTVSS	0.67/2.01	P48R, H86R	0.278/0.171	T11M, T19M, T36, T54M, T62M, T63M, T73M, T82M, T95M, T120M	0.683	0.548	76.36	62.64
VH-0743-166-7	SVQLVESGGGLVQPGGSLRLSCAA**SGVNINSNG**GRVAWVRQAPGKGLEWVSSISHDGGETTIADSVKGRFTISRDNSKNTLYLQMNSLRAEDTAVYYCARVDNSGKPYHGGQGTTVTVSS	0.96/1.89	L47R, I62Y, A90P, V101R, T115P	0.377/0.304	C22E/K, C98E/K, N28M, N30M, N32M, N76M, N79M, N86M	0.783	0.708	96.03	62.34
VH-0743-166-7H	EVQLVESGGGLVQPGGSLRLSCAAS**GVNINSNGG**RVAWVRQAPGKGLEWVSSISHDGGETTYADSVKGRFTISRDNSKNTLYLQMNSLRAEDTAVYYCARVDNSGKPYHWGQGTLVTVSS	1.09/1.94	No mutations proposed	0.376/0.27	C22L, C98L, N28L, N30L, N32L, N76L, N79L, N86L, N103L, Y62L	0.831	0.754	98.47	62.45

N/A—Not attempted. ^1^ CDR-loops for native nanobodies are provided as designed by RFantibody; CDR-loops of humanized nanobodies are defined by the Chothia CDR definition on the NB2 webserver; the CDR-loop definition within NB2 is identical for both native and humanized structures. ^2^ Intrinsic/structurally corrected. ^3^ Mutations with stability and solubility rank 1 are proposed. ^4^ Native/considering top-10 single-point mutations. Top-score mutations are highlighted in bold. ^5^ Top-10 mutations, if available, for each structure are proposed. Mutations are considered to be single-point, unlike the CamSol Comb. ones.

## Data Availability

Instructions to reproduce steps or extrapolate the method of the de novo nanobody design pipeline and the intermediate reports of results described in this paper are available in the GitHub repository, https://github.com/danilkotelnikov/NanoDeNovo.git (accessed on 12 July 2025). Due to the huge dataset size, only production results are shared publicly but can be requested at any moment by email or opening a GitHub issue.

## Supplementary Materials

The following supporting information can be downloaded at https://www.mdpi.com/article/10.3390/ijms26199262/s1.
