# Supplementary material for: NanoDeNovo: De Novo Design of Anti-Poliovirus I Sabin Strain Nanobodies by Semi-Automated Computational Pipeline
Source: Int J Mol Sci. 2025 Sep 23;26(19):9262. doi: 10.3390/ijms26199262 (PMC12524548; doi:10.3390/ijms26199262)
Supplement: Supplementary file 1 [file ijms-26-09262-s001.zip › ijms-3828781-supplementary.pdf]

SUPPLEMENTARY FILE

NanoDeNovo: De Novo Design of Anti-Poliovirus I Sabin Strain Nanobodies by Semi-Automated Computational Pipeline

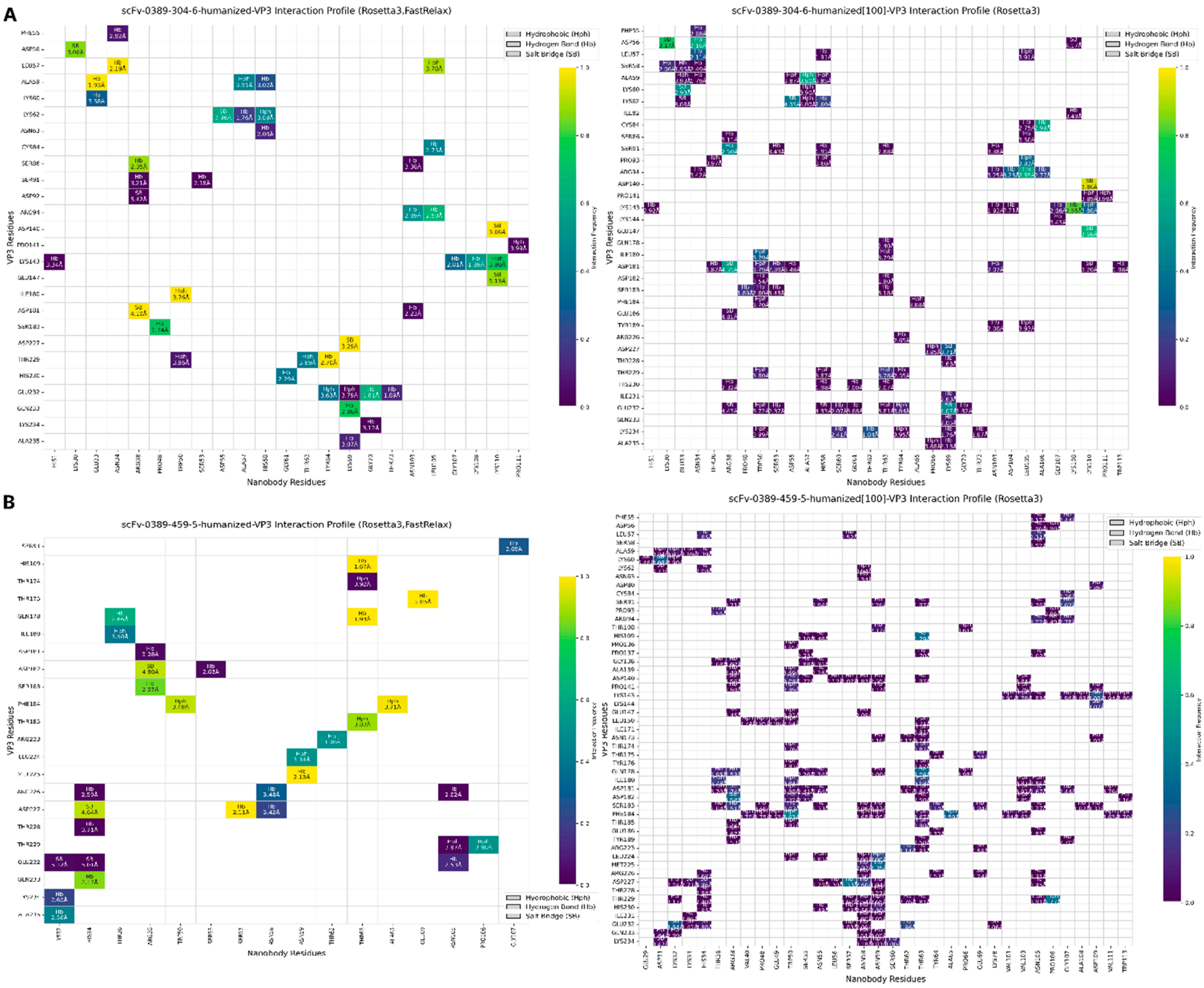

Figure S1. Interaction profiles of humanized scFv-0389-304-6 (**A**) and scFv-0389-459-5 (**B**) nanobodies. ScFv-0389-304-6H heatmaps show that predominant amounts of interactions repeat native-like binding pairs (rate >0.4) and all possible local minimas from the top-scored complex. ScFv-0389-459-5H have lost its former consistency among Rosetta3 global docking predictions, resulting in a huge amount of scattered poses and multiple favourable binding modes.

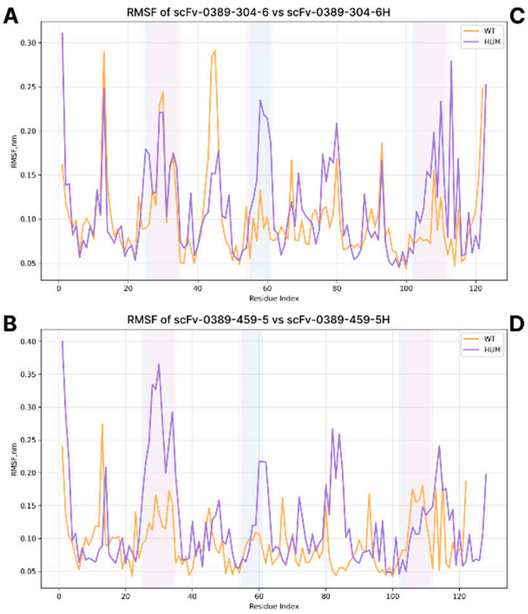

C

scFv-0389-304-6

Energy Component	Average	SD(Prop.)	SD	SEM(Prop.)	SEM
ΔBOND	-0.00	9.61	0.00	0.30	0.00
ΔANGLE	-0.00	15.35	0.00	0.49	0.00
ΔDIHED	0.00	7.98	0.00	0.25	0.00
ΔVDWAALS	-127.32	6.73	13.23	0.21	0.42
ΔEEL	-232.41	26.16	58.17	0.83	1.84
Δ1-4 VDW	0.00	6.16	0.00	0.19	0.00
Δ1-4 EEL	-0.00	19.30	0.00	0.61	0.00
ΔEPB	302.26	25.71	56.79	0.81	1.80
ΔENPOLAR	-12.98	0.20	1.16	0.01	0.04
ΔEDISPER	0.00	0.00	0.00	0.00	0.00
ΔGGAS	-359.73	27.01	62.46	0.85	1.97
ΔGSOLV	289.28	25.71	56.24	0.81	1.78
**ΔTOTAL**	-70.45	37.29	12.44	1.18	0.39

scFv-0389-304-6H

Energy Component	Average	SD(Prop.)	SD	SEM(Prop.)	SEM
ΔBOND	-0.00	9.76	0.00	0.31	0.00
ΔANGLE	-0.00	14.05	0.00	0.44	0.00
ΔDIHED	-0.00	08.07	0.00	0.26	0.00
ΔVDWAALS	-64.21	1.17	14.73	0.04	0.47
ΔEEL	-243.45	17.27	65.68	0.55	2.8
Δ1-4 VDW	0.00	5.55	0.00	0.18	0.00
Δ1-4 EEL	0.00	19.43	0.00	0.61	0.00
ΔEPB	277.86	7.42	67.30	0.23	2.13
ΔENPOLAR	-7.85	0.85	1.57	0.03	0.05
ΔEDISPER	0.00	0.00	0.00	0.00	0.00
ΔGGAS	-307.67	17.31	72.45	0.55	2.29
ΔGSOLV	270.01	7.47	66.62	0.24	2.11
**ΔTOTAL**	-37.66	18.85	10.35	0.60	0.33

D

scFv-0389-459-5

Energy Component	Average	SD(Prop.)	SD	SEM(Prop.)	SEM
ΔBOND	-0.00	10.45	0.00	0.33	0.00
ΔANGLE	0.00	15.28	0.00	0.48	0.00
ΔDIHED	-0.00	9.35	0.00	0.30	0.00
ΔVDWAALS	-86.96	9.65	10.31	0.31	0.33
ΔEEL	-297.31	44.82	32.35	1.42	1.2
Δ1-4 VDW	0.00	5.80	0.00	0.18	0.00
Δ1-4 EEL	-0.00	21.43	0.00	0.68	0.00
ΔEPB	326.36	37.68	34.60	1.19	01.09
ΔENPOLAR	-9.14	0.57	0.78	0.02	0.02
ΔEDISPER	0.00	0.00	0.00	0.00	0.00
ΔGGAS	-384.27	45.85	35.20	1.45	1.11
ΔGSOLV	317.22	37.69	34.10	1.19	01.08
**ΔTOTAL**	-67.05	59.35	10.92	1.88	0.35

scFv-0389-459-5H

Energy Component	Average	SD(Prop.)	SD	SEM(Prop.)	SEM
ΔBOND	-0.00	10.35	0.00	0.33	0.00
ΔANGLE	0.00	13.96	0.00	0.44	0.00
ΔDIHED	-0.00	8.61	0.00	0.27	0.00
ΔVDWAALS	-67.95	6.17	31.66	0.19	1.00
ΔEEL	-196.41	46.47	99.26	1.47	3.14
Δ1-4 VDW	-0.00	4.99	0.00	0.16	0.00
Δ1-4 EEL	0.00	21.39	0.00	0.68	0.00
ΔEPB	231.43	30.61	113.25	0.97	3.58
ΔENPOLAR	-7.18	1.10	3.31	0.03	0.10
ΔEDISPER	0.00	0.00	0.00	0.00	0.00
ΔGGAS	-264.36	46.88	128.60	1.48	4.6
ΔGSOLV	224.25	30.63	110.13	0.97	3.48
**ΔTOTAL**	-40.11	56.00	20.01	1.77	0.63

Figure S2. A-B. Comparison of RMSF between native (WT) and humanized (HUM) nanobodies over 100-ns molecular dynamics simulations. Overall analysis shows that fluctuations are within ranges of <0.5-3Å for scFv-0389-304-6 and <0.5-4Å for scFv-0389-459-5. CDR regions of WT and HUM nanobodies are highlighted with pale blue and red colors on the background. **C-D.** MM-PBSA free energy calculation (Δ(Complex − Ligand − Receptor)) results of WT and humanized complexes calculated over 100-ns (1000 frames) with Uni-GBSA resulted in significant yet acceptable values.

.

Table S1. RFantibody design statistics.

Scaffold	Design IDs	ipAE	pAE	Framework-aligned RMSD of, Å					Mean dG_cross[1]	ANARCII score
				Nanobody	CDRs	H1	H2	H3		
ScFv-0743	166-7	2.36	3.53	0.81	1.38	1.82	0.65	1.13	-50.18	29.13

[1] Calculated over 10 FastRelaxed complexes for each scaffold.

Table S2. Molecular docking cross-validation of redesigned scFv-0743-166-7 nanobody.

Nanobody/ Scaffold	Number of total complexes/clusters		Mean interface score for Rosetta3[1]	Rosetta3 CAPRI rank mode[1]	Mean interface score for RD2	RD2 CAPRI rank mode[1]	Lowest CP2 score[2]/ cluster rank	Highest CP2 DockQ score (classification)
	Rosetta3	CP2						
ScFv-0743-166-7	6810	30	-23.02	3	-22.36	3	-173.1/12	0.4183 (acceptable)

[1] Calculated over top-100 complexes, ranked by I_sc; in this particular case an input complex served as a reference for CAPRI calculation.

[2] Taken for the complex with the best DockQ compared to the FastRelaxed complex; for humanized nanobodies we picked the best score possible considering both NB2 and Chai-1 structures.

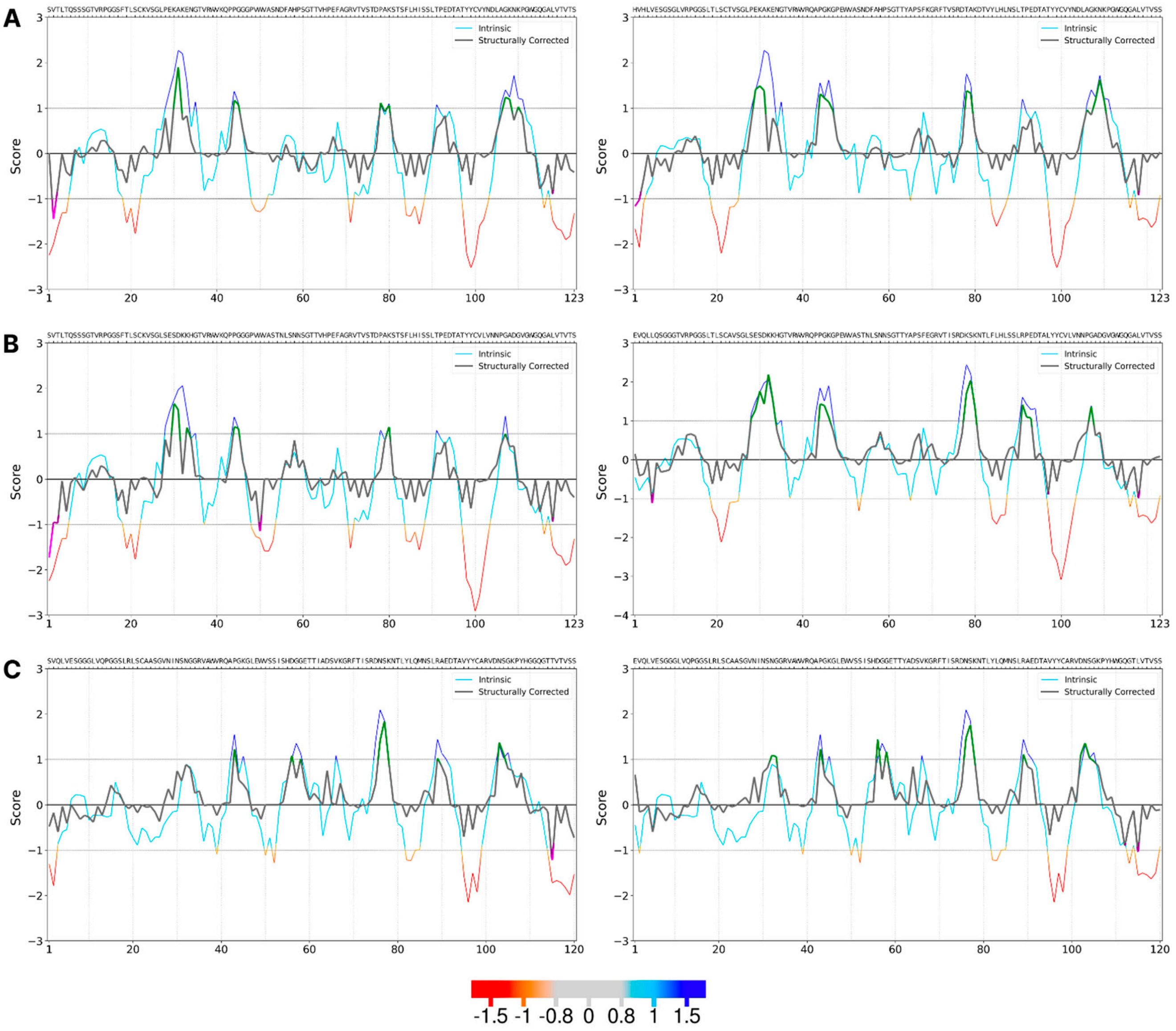

Figure S3. Solubility profile plots of scFv-0389-304-6/H (**A**), scFv-0389-459-5/H (**B**), and scFv-0743-166-7/H (**C**) nanobodies built by CamSol Intrinsic (sequence-based) and Structurally Corrected methods with a bar indicating the relative solubility score. Values ≤1 indicate poorly soluble regions, while values ≥1 indicate highly soluble regions.

.

Table S3. Physicochemical properties of de novo designed nanobodies predicted by ProtParam tool.

Nanobody ID	Amino Acids	Molecular Weight (Da)	Theoretical pI	Ext. Coeff. (M^{-1} cm^{-1})	Abs 0.1% (1 g/L)	Half-life (hours)[1]	Instability Index	Aliphatic Index	GRAVY
scFv-0389-304-6	123	12811.27	8.64	21095	1.647	1.9	38.67	62.52	-0.303
scFv-0389-304-6H	123	13109.62	8.62	24075	1.836	3.5	29.13	67.32	-0.404
scFv-0389-459-5	123	12623.98	7.80	19605	1.633	1.0	34.95	68.78	-0.185
scFv-0389-459-5H	123	12919.41	8.72	21095	1.888	1.0	37.90	77.56	-0.307
scFv-0743-166-7	120	12518.83	8.68	17085	1.553	1.9	29.82	74.67	-0.342
scFv-0743-166-7H	120	12752.10	8.07	24075	1.365	1.9	30.52	74.67	-0.380

[1] Predicted value for mammalian reticulocytes, in vitro.

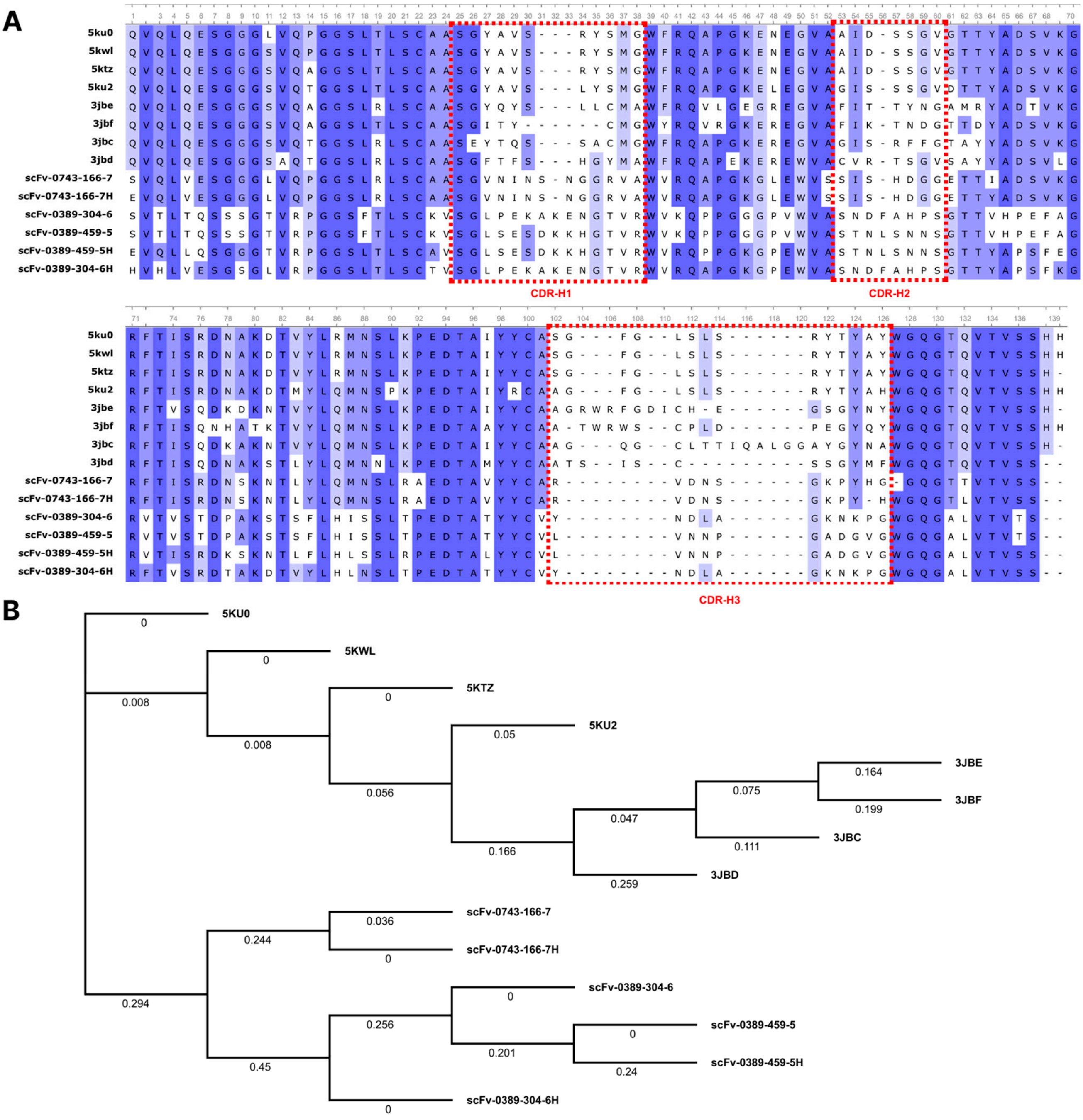

Figure S4. A. Multiple sequence alignment performed with the MUSCLE algorithm shows a very high conservation level between in silico and in vitro designed nanobodies specific to VP3. Highlighting of characters is set at ≥50% conservation level. ScFv-0743-166/H design establishes distinct similarities with real structures in CDR regions at 35 (G), 54 (I), 60 (G) positions. **B.** A phylogenetic tree (viewed as a cladogram) built by IQ-TREE depicts several nanobody clusters with similarity distances beneath the lines. ScFv-0743-166-7/H nanobodies possess the least evolutionary distance compared to real structures.

Percent Identity Matrix

scFv-0389-304-6	100.00%	79.67%	83.74%	64.23%	45.00%	45.83%	37.82%	39.32%	38.66%	38.79%	42.86%	43.70%	39.50%	43.70%
scFv-0389-304-6H	79.67%	100.00%	63.41%	68.29%	58.33%	60.83%	51.26%	50.43%	49.58%	51.72%	57.98%	59.66%	53.78%	58.82%
scFv-0389-459-5	83.74%	63.41%	100.00%	80.49%	45.83%	46.67%	38.66%	41.88%	40.34%	38.79%	43.70%	44.54%	41.18%	44.54%
scFv-0389-459-5H	64.23%	68.29%	80.49%	100.00%	59.17%	62.50%	50.42%	52.14%	49.58%	49.14%	54.62%	55.46%	52.10%	55.46%
scFv-0743-166-7	45.00%	58.33%	45.83%	59.17%	100.00%	96.67%	61.02%	61.54%	58.47%	58.26%	61.86%	63.56%	63.56%	62.71%
scFv-0743-166-7H	45.83%	60.83%	46.67%	62.50%	96.67%	100.00%	62.71%	63.25%	60.17%	60.00%	63.56%	65.25%	65.25%	64.41%
3jbc	37.82%	51.26%	38.66%	50.42%	61.02%	62.71%	100.00%	72.50%	74.02%	72.95%	73.98%	73.17%	75.61%	73.98%
3jbd	39.32%	50.43%	41.88%	52.14%	61.54%	63.25%	72.50%	100.00%	66.95%	68.97%	68.33%	67.50%	69.17%	68.33%
3jbe	38.66%	49.58%	40.34%	49.58%	58.47%	60.17%	74.02%	66.95%	100.00%	72.50%	70.25%	68.60%	68.60%	69.42%
3jbf	38.79%	51.72%	38.79%	49.14%	58.26%	60.00%	72.95%	68.97%	72.50%	100.00%	73.11%	71.67%	70.83%	72.50%
5ktz	42.86%	57.98%	43.70%	54.62%	61.86%	63.56%	73.98%	68.33%	70.25%	73.11%	100.00%	98.37%	90.24%	99.19%
5ku0	43.70%	59.66%	44.54%	55.46%	63.56%	65.25%	73.17%	67.50%	68.60%	71.67%	98.37%	100.00%	89.52%	99.19%
5ku2	39.50%	53.78%	41.18%	52.10%	63.56%	65.25%	75.61%	69.17%	68.60%	70.83%	90.24%	89.52%	100.00%	90.32%
5kwl	43.70%	58.82%	44.54%	55.46%	62.71%	64.41%	73.98%	68.33%	69.42%	72.50%	99.19%	99.19%	90.32%	100.00%

Figure S5. Percent identity matrix built via UniProt MSA server utilizing ClustalO algorithm depicting almost all humanized nanobodies exceeding 50% similarity, compared to real structures. Except for the 3JBF and 3JBE, ScFv-0743-166-7/H designs possess similarities even at higher values, reaching ≥60% identity.

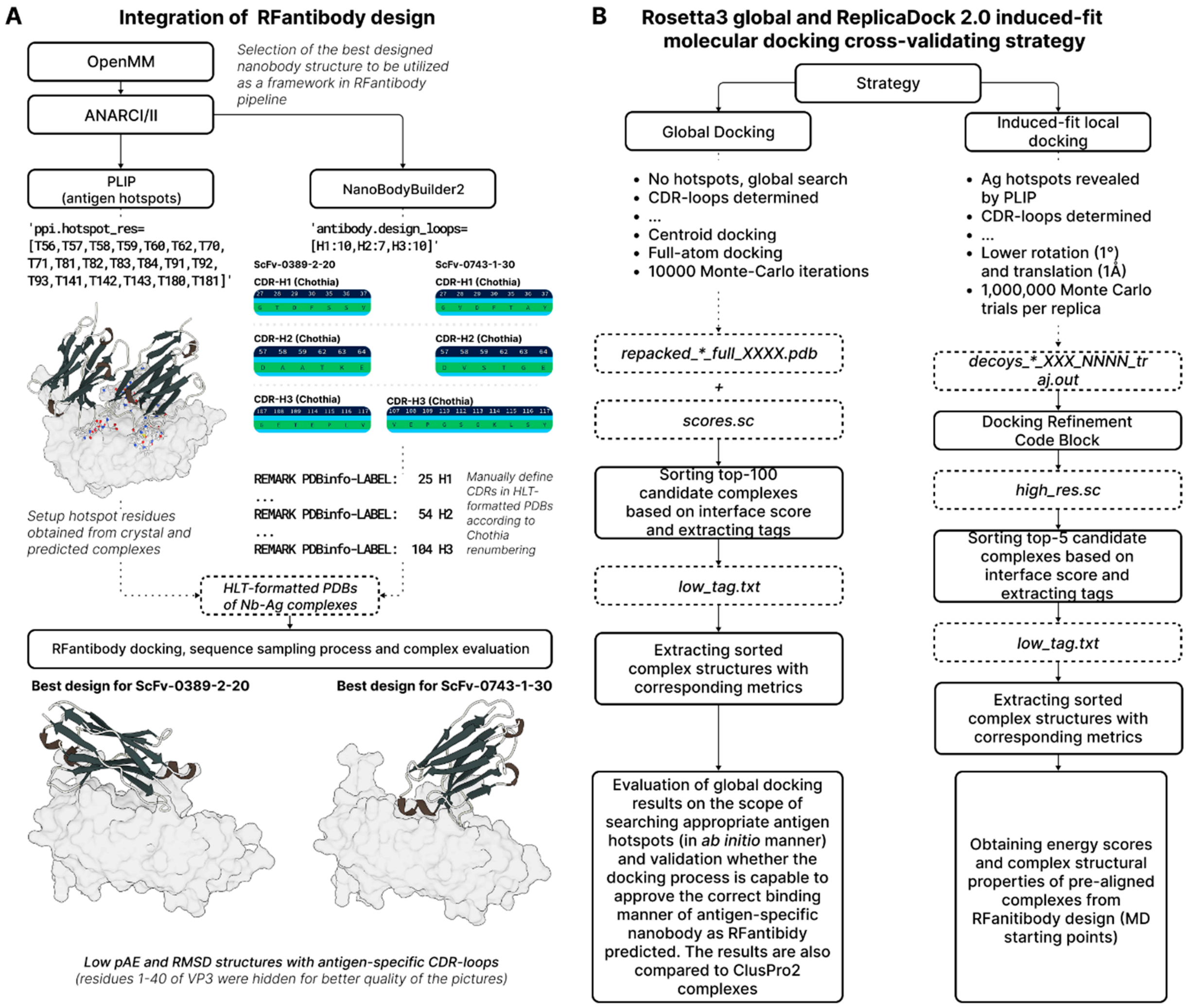

Figure S6. A. Graphical abstract of RFantibody integration and proper parametrization for nanobody and antibody design. **B.** Graphical abstract of custom Rosetta ReplicaDock 2.0 pipeline validation strategy for de novo design.

.

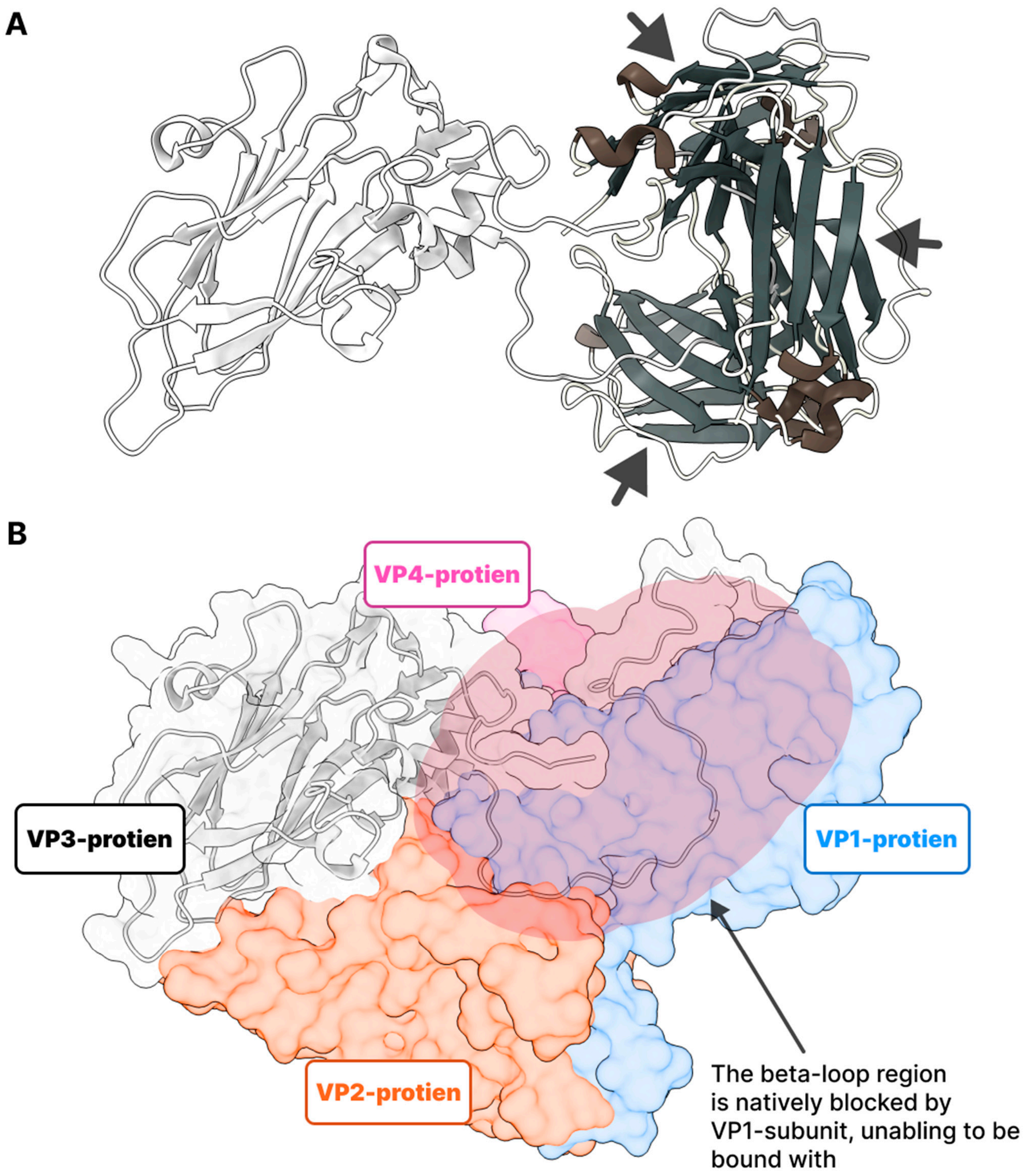

Figure S7. A. Nanobodies pointed with errors are misbound and trapped in the β-loop caveat. **B.** Original structure of Poliovirus I Sabin strain VP-complex (PDB-ID: 8E8Z) shows that the caveat region (shown with cartoon and highlighted with red) is natively blocked by VP-1 subunit. Blue — VP1-protein, orange — VP2-protein, light grey — VP3-protein, pink — VP4-protein.
